# Biomimetic chameleon soft robot with artificial crypsis and disruptive coloration skin

**DOI:** 10.1038/s41467-021-24916-w

**Published:** 2021-08-10

**Authors:** Hyeonseok Kim, Joonhwa Choi, Kyun Kyu Kim, Phillip Won, Sukjoon Hong, Seung Hwan Ko

**Affiliations:** 1 Applied Nano and Thermal Science Lab, Department of Mechanical Engineering, Seoul National University, Seoul, Korea; 2 Optical Nanoprocessing Lab, Department of Mechanical Engineering, BK21 FOUR ERICA-ACE Center, Hanyang University, Ansan, Gyeonggi-do Korea; 3 Institute of Advanced Machinery and Design (SNU-IAMD)/Institute of Engineering Research, Seoul National University, Seoul, Korea

**Keywords:** Mechanical engineering, Materials for devices, Optical materials and structures

## Abstract

Development of an artificial camouflage at a complete device level remains a vastly challenging task, especially under the aim of achieving more advanced and natural camouflage characteristics via high-resolution camouflage patterns. Our strategy is to integrate a thermochromic liquid crystal layer with the vertically stacked, patterned silver nanowire heaters in a multilayer structure to overcome the limitations of the conventional lateral pixelated scheme through the superposition of the heater-induced temperature profiles. At the same time, the weaknesses of thermochromic camouflage schemes are resolved in this study by utilizing the temperature-dependent resistance of the silver nanowire network as the process variable of the active control system. Combined with the active control system and sensing units, the complete device chameleon model successfully retrieves the local background color and matches its surface color instantaneously with natural transition characteristics to be a competent option for a next-generation artificial camouflage.

## Abstract (web summary)

Realizing an artificial camouflage device with a high spatial resolution by adapting to the surrounding environment in real-time is a challenging task, mainly associated with device fabrication and integration with sensor and control circuits. To overcome these limitations, the authors utilize thermochromic liquid crystal ink that reacts to the feedback control system of the vertically stacked silver nanowire heater.

## Introduction

Artificial camouflage is the functional mimicry of the natural camouflage that can be observed in a wide range of species^1–3^. Especially, since the 1800s, there were a lot of interesting studies on camouflage technology for military purposes which increases survivability and identification of an anonymous object as belonging to a specific military force^4,5^. Along with previous studies on camouflage technology and natural camouflage, artificial camouflage is becoming an important subject for recently evolving technologies such as advanced soft robotics^1,6–8^ electronic skin in particular^9–12^. Background matching and disruptive coloration are generally claimed to be the underlying principles of camouflage covering many detailed subprinciples^13^, and these necessitate not only simple coloration but also a selective expression of various disruptive patterns according to the background. While the active camouflage found in nature mostly relies on the mechanical action of the muscle cells^14–16^, artificial camouflage is free from matching the actual anatomies of the color-changing animals and therefore incorporates much more diverse strategies^17–22^, but the dominant technology for the practical artificial camouflage at visible regime (400–700 nm wavelength), especially RGB domain, is not fully established so far. Since the most familiar and direct camouflage strategy is to exhibit a similar color to the background^23–25^, a prerequisite of an artificial camouflage at a unit device level is to convey a wide range of the visible spectrum that can be controlled and changed as occasion demands^26–28^. At the same time, the corresponding unit should be flexible and mechanically robust, especially for wearable purposes, to easily cover the target body as attachable patches without interrupting the internal structures, while being compatible with the ambient conditions and the associated movements of the wearer^29,30^.

System integration of the unit device into a complete artificial camouflage device, on the other hand, brings several additional issues to consider apart from the preceding requirements. Firstly, the complexity of the unit device is anticipated to be increased as the sensor and the control circuit, which are required for the autonomous retrieval and implementation of the adjacent color, are integrated into a multiplexed configuration. Simultaneously, for nontrivial body size, the concealment will be no longer effective with a single unit unless the background consists of a monotone. As a simple solution to this problem, unit devices are often laterally pixelated^12,18^ to achieve spatial variation in the coloration. Since its resolution is determined by the numbers of the pixelated units and their sizes, the conception of a high-resolution artificial camouflage device that incorporates densely packed arrays of individually addressable multiplexed units leads to an explosive increase in the system complexity. While on the other hand, solely from the perspective of camouflage performance, the delivery of high spatial frequency information is important for more natural concealment by articulating the texture and the patterns of the surface to mimic the microhabitats of the living environments^31,32^. As a result, the development of autonomous and adaptive artificial camouflage at a complete device level with natural camouflage characteristics becomes an exceptionally challenging task.

Our strategy is to combine thermochromic liquid crystal (TLC) ink with the vertically stacked multilayer silver (Ag) nanowire (NW) heaters to tackle the obstacles raised from the earlier concept and develop more practical, scalable, and high-performance artificial camouflage at a complete device level. The tunable coloration of TLC, whose reflective spectrum can be controlled over a wide range of the visible spectrum within the narrow range of temperature^33,34^, has been acknowledged as a potential candidate for artificial camouflage applications before^21,34^, but its usage has been more focused on temperature measurement^35–38^ owing to its high sensitivity to the temperature change. The susceptible response towards temperature is indeed an unfavorable feature for the thermal stability against changes in the external environment, but also enables compact input range and low power consumption during the operation once the temperature is accurately controlled.

The selection of an appropriate heater together with a proper control circuit is therefore critical for the development of a TLC-based artificial camouflage device, and we conclude that Ag NW heater enables not only accurate temperature manipulation but also a different route to express the fine patterns without associating the lateral pixelation scheme mentioned earlier. Owing to its superior electrical and mechanical stability, Ag NW has been a promising material for flexible^39,40^ and stretchable heaters^41,42^. Also, comparing with other materials such as gold (Au) NW, Copper (Cu) NW^43^, and hybrid materials (Cu-Ni NW^44^, Ag-Au NW^45^, and Ag NW-CNT^46^, Ag NW-PEDOT:PSS^47^), Ag NW has excellent electrical conductivity and oxidation resistance as a single material and has a cost-effective feature to be applied for large-area applications through a simple synthesis process. While at the same time, we noticed that the temperature coefficient of resistance (TCR) of the Ag NW network is sufficiently large, linear, and non-hysteric. These properties led us to use the resistance of Ag NW network as the process variable of a negative feedback control system to maintain the target temperature under external environment fluctuations. The active control of the heat flux also permits a further reduction in the response time even to be comparable to the physiological color change found in animals^48^.

Meanwhile, the evolution of polymorphism in specific species^49^ suggests that the display of an arbitrary image, which is only conceivable via high resolution, individually addressable lateral pixels, is superfluous for many camouflage applications that are subject to the limited number of habitats^31,32,50^. In this regard, instead of constructing innumerable miniaturized lateral heaters, Ag NW heaters are firstly laser-patterned to the selected habitats and then piled vertically in a multilayer configuration. By stacking the Ag NW heaters composed of largely void and negligible thickness, the temperature profiles generated by the distinct heaters are superposed at the outermost TLC layer to allow the matching of the background color and the expression of the microhabitat at the same time. The corresponding strategy allows a great reduction in the overall system complexity compared to the previous approaches together with more natural camouflage characteristics by eliminating the dead zone between pixels and assisting acute but continuous transition in the coloration. At last, by integrating the proposed Ag NW and TLC-based Artificial Chameleon Skin (ATACS) with color sensors and feedback control systems, adaptive artificial camouflage at a complete version of the device which is capable of detecting the local background color and matching its coloration in real-time has been accomplished on a chameleon model. Large-area, the natural and rapid coloration of the moving chameleon according to the underlying habitat grants the potential of the proposed scheme as a scalable and practical next-level camouflage technology.

## Results

### Vertically stacked ATACS consisted of Ag NW heaters and TLC

**Fig. 1:**
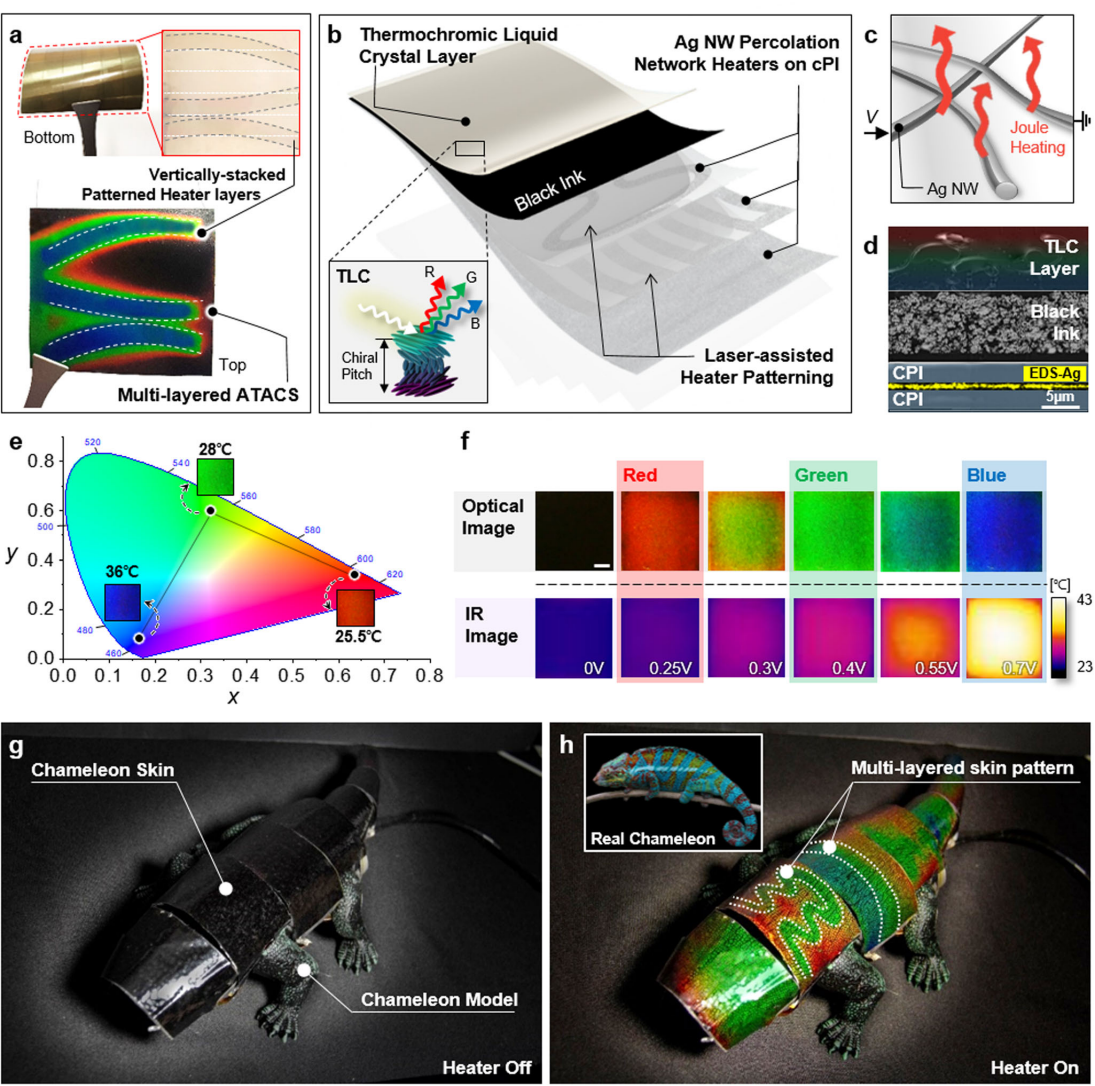
Multi-layered ATACS capable of generating multiple skin patterns with RGB coloration. **a** Digital image of Multi-layered ATACS and its bottom image with vertically stacked patterned heater layers. **b** Configuration of the multi-layered ATACS: TLC is coated on the black ink layer. Ag NWs percolation network heaters were stacked on cPI films and patterned by the UV laser ablation process. **c** The Ag NW heater generates heat with a joule heating mechanism. **d** SEM image of the cross-section of its EDS image for silver. The scale bar is 5 µm. **e** CIE 1931 chromaticity diagram of the ATACS and actual image of it at 25.5, 28, and 36 °C. **f** Coloring performance of the ATACS depending on various DC voltage. The scale bar is 1 mm. **g** The chameleon model with 7 of the multi-layered highly flexible ATACS patches. **h** Demonstration of the multi-layered ATACT patches on the chameleon model. Inset is a real chameleon with full coloring.

TLC ink layer and an Ag NW heater, or multiple Ag NW heaters, are the core elements of the proposed multi-layered ATACS. For a typical ATACS, the vertically stacked patterned heater layers of the multi-layered ATACS are observable from the bottom. In Fig. 1a, two heater layers with straight stripes and the wavy patterns are visible from the optical image as translucent ribbons. The upper TLC ink layer exhibits vivid color in the visible regime upon the activation of these vertically stacked heaters. The optical image of the multi-layered ATACS under operation captured from the top verifies that the pattern from the selected heater, i.e., the one with the wavy pattern in this demonstration, is transferred to the outermost TLC ink layer, notably independent to the presence of other heater layers.

The overall structure of the multi-layered ATACS is illustrated in Fig. 1b and Supplementary Fig. 1. The coloration by the outermost TLC ink stems from the helical texture that selectively reflects a specific light, and the cholesteric pitch, hence the reflected spectrum, can be manipulated in a wide range of the visible spectrum by a single parameter: temperature^33^ (detail description in Supplementary Note 1). The underlying heater is composed of randomly distributed Ag NWs^41^ on colorless polyimide (cPI) substrate (Fig. 1c) for stable thermal characteristics^51^, and the number of the Ag NW heaters, either patterned by laser or non-patterned, varies according to the number of target microhabitats. Since ATACS is based on TLC which operates under elevated temperature and its substrate should have the capability of soft feature to apply soft robots, cPI is one of the great substrate materials with high-temperature stability and mechanical flexibility. Also, The Ag NW heaters are recognized to be highly flexible^52^, stable under mechanical deformations^53^, and easily patternable^54^ from the previous studies, and it is worth mentioning in addition that the entire ATACS including the TLC layer and the readout electrode is produced through a series of wet processing in ambient condition (Supplementary Fig. 2 for the detailed fabrication steps) to secure large-area applicability. The cross-sectional scanning electron microscopy (SEM) image of the resultant ATACS with a single Ag NW heater (Fig. 1d) indicates that the total thickness is only ~30 µm. Multiple Ag NW heaters can be integrated likewise without losing the overall flexibility since the thickness occupied by a single cPI-Ag NW combination is less than 10 µm, whereas this value can be reduced further by thinning the cPI layer. Due to the thickness of the Ag NW layer in each Ag NW heater is negligible (~100 nm), reducing the thickness of the cPI layer is important to realize a highly flexible ATACS^55^. For a more vibrant reflective color, black ink is inserted between the TLC layer and Ag NW heater to absorb the remaining spectrum that is not subject to the Bragg reflection.

The temperature at the TLC layer determines the reflective spectrum of the ATACS, and three points correspond to the surface temperature of 25.5, 28, and 36 °C are plotted in the CIE 1931 graph as representative RGB colors (Fig. 1e). The optical photographs and the corresponding IR images according to the input voltage shows that the coloration of the TLC layer possesses one-to-one correspondence to the surface temperature with continuous transition characteristics that enable a variety of colors with a single input parameter (Fig. 1f and Supplementary Fig. 3). The input range, in terms of both surface temperature and applied voltage, can be adjusted to meet the requirements of the operating conditions by changing the compositions of the TLC layer^34^ or the concentration of the constituent Ag NW^56^. For the current study, the onset temperature for the coloration is set to be in proximity to the ambient temperature (*T*_amb_ = 20 °C) in order to minimize the power consumption required for the active camouflage. Under the current conditions and the constituent materials, the power required to maintain blue coloration is estimated to be 19.4 mW/cm^2^, allowing a single commercial AA battery (2500 mAh, 1.2 V) to maintain the active camouflage at 10 × 10 cm^2^ area for 92 mins. Together with the low power consumption, the stability of an ATACS is further tested through an on-off cyclic test. (Supplementary Fig. 4).

A highly flexible ATACS permits the concealment of the target body by attaching the corresponding units as patches. 7 distinct patches ranging from 21.96 to 67.2 cm^2^ in relation to the shape of the chameleon model are prepared and attached conformally on the surface to fully cover the exterior as shown in Fig. 1g. Also, for thermal insulation with the chameleon body, a spacer is placed on the chameleon robot body to secure the air gap between the robot body and ATACS (Supplementary Fig. 28). The color exhibited by the chameleon is black when the surface temperature is at the ambient temperature, yet the vibrant color is observable upon the activation of the underlying heaters to reach the operating temperature range (Fig. 1h). Since each ATACS patch is regarded as a single pixel, the delivery of fine patterns is smaller than the patch size, e.g., the stripe patterns found in the real chameleon in the inset of Fig. 1h, cannot be achieved with a single uniform heater. Therefore, 3 distinct Ag NW heaters—uniform, stripe, wavy—are vertically integrated with each ATACS to exhibit fine patterns together with the designated background color as representatively shown in the two pixels in the middle of the chameleon. The Ag NW heaters ablated at the stripe and the wavy patterns are operated in each pixel while both accompanied by the uniform heater, to create discernable patterns together with a background color to match the overall terrain and the microhabitat simultaneously.

### Performance of the ATACS

**Fig. 2:**
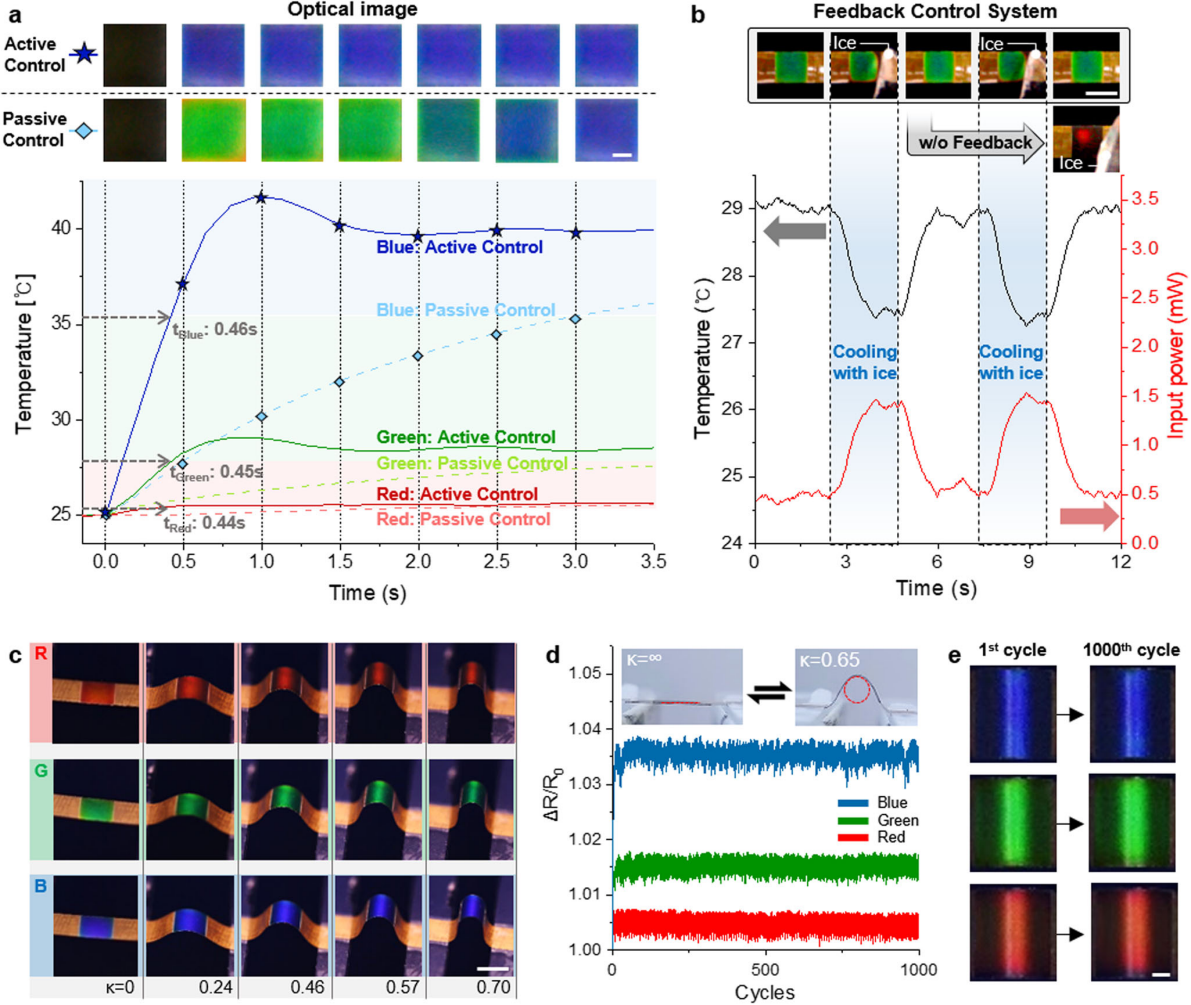
The ATACS with fast and stable controllability, and its performance under mechanical deformation. **a** Temperature response profiles of the ATACS with active control and passive control. The top images are the digital image of the ATACS with respect to time. The scale bar is 2 mm. **b** Optical images of the ATACS with a feedback control system under cooling with ice. A graph of the feedback control system operation under temperature drop of the ATACS. The scale bar is 5 mm. **c** Digital image of RGB colored ATACS with various curvatures. The scale bar is 5 mm. **d** Electrical resistance variation of the ATACS during a long-term cyclic test over 1000 times bending. **e** ATACS without degradation in color performance after 1000 cycles of repeated bending. The scale bar is 1 mm.

Two potential drawbacks of a thermochromic camouflage are the limited response speed and low stability towards external environment change, and we confirm that the active control of the input voltage in accordance with the status of the ATACS through the control circuit can provide an efficient solution to these problems. Instead of applying a constant bias voltage, the input voltage is actively modulated to reduce the characteristic time from 3.04, 3.44, and 3.52 s (passive control) to 0.44, 0.45, and 0.46 s (active control) for RGB signals respectively, incorporating minor overshoot before it reaches the steady-state temperature (Fig. 2a). Even in the lower surrounding temperature (15 °C, 5 °C, −5 °C), the ATACS shows short characteristic times <0.6 s (Supplementary Fig. 5). The ATACS under active control, having comparable timescale to a physiological response^14,16^, enables a practically instantaneous shift in the coloration to the human eye as verifiable from the real-time demonstration of RGB colors (Supplementary Movie 1). The passive control also fails the ATACS from adapting itself to the external environmental change, such as a sudden decrease in the surrounding temperature. For robust preservation of the designated color, the input power is actively modulated by the PID control-based feedback system (Supplementary Fig. 6 for detailed information) in accordance with the temperature of the TLC layer, which is estimated from the TCR of the Ag NW heater (Supplementary Fig. 7). The strengthened stability under the feedback system is tested by bringing a piece of ice in proximity to the ATACS, which lowers the surrounding temperature substantially. As the temperature of the TLC layer drops, the input power is increased accordingly under the operation of the feedback system in order to compensate for the heat loss and recover the target temperature (Fig. 2b). The drastic difference in the stability is observable from the ATACS in the inset image, which shows severe color change under the identical environmental disturbance without the feedback system (Supplementary Fig. 8 and Supplementary Movie 2 for the real-time demonstration of the ATACS with and without the feedback system).

The commercial TLC used in this study changes its color in the temperature range of 25.5 °C to 36 °C, and since the Ag NW percolation network only enables heating mode, the expressible spectrum will be limited when the ambient temperature is over 25.5 °C (Supplementary Fig. 9). Also, it is difficult to have a uniform color distribution over the entire area at an extremely low −20 °C due to the inherent limitation of TLC, in which the color changes due to temperature. Through the control, it is possible to control the average temperature of the entire area of ATACS, but it shows a steep temperature gradient at extremely low temperatures (Supplementary Fig. 11). As a future study to break these limitations, we can consider two different solutions regarding this matter: (i) The operating temperature range of a TLC can be shifted by changing its chemical composition^57^. The working temperature range for commercially available TLC mixtures spans from −30 °C to 115 °C^57^. In this sense, the TLC material can be selected in accordance with the ambient temperature of the target area. (ii) By substituting the Ag NW heater with a thermoelectric device, both heating and cooling modes can be achieved; however, since most of the flexible thermoelectric device relies on rigid thermoelectric pillars^58,59^, it is difficult to create the proposed vertically stacked configuration.

Since the accomplishment of the feedback system relies on the temperature-dependent electrical resistance change of the Ag NW heater as the process variable, its invariance to the mechanical, chemical stimuli has to be ensured in advance. The Ag NW heater shows excellent mechanical stability and chemical stability as well because of the cPI cover layer (Supplementary Figs. 12–14). Also, the maximum allowable mechanical deformation is determined by the curvature of the ATACS, and Fig. 2c confirms that each coloration at RGB can be retained at least up to the curvature of 0.70 mm^−1^ under constant input voltage without showing noticeable alteration in its coloration, approving that the electrical resistance change due to the mechanical bending is relatively small (Supplementary Fig. 15). The current curvature range is expected to be sufficient for many cases, especially for large-area camouflage applications, e.g., for the chameleon model employed in this study. While at the same time, in the actual operation, the target body under concealment may incorporate continuous movement, which in turn requires dynamic stability of the attached ATACS over an extended period. The cyclic bending test at *κ* = 0.65 mm^−1^ in Fig. 2d confirms that the change in the normalized electrical resistance is only within 0.5 % for the complete 1000 cycles without any permanent change in the R_0_ value. Since the variation in electrical resistance due to the mechanical bending is clearly distinguishable from the ones that arise from coloration, the feedback control remains effective even under mechanical bending. In addition, the ATACS shows stable color performance during and after 1000 cycles without any hysteresis (Fig. 2e). It is also worth mentioning that the sensitivity of the electrical resistance towards mechanical disturbance can be further reduced through the alteration of the Ag NW density of the percolation network^56^ (Supplementary Fig. 17).

### Effective camouflage of ATACS with multi-patterned heater

Given that the expression of microhabitat features through the laterally pixelated scheme can be extremely challenging especially with the individual sensing unit and feedback system as mentioned earlier, we bring a more conceivable solution for the expression of fine patterns from the polymorphism found in nature^60^. The simultaneous matching of background color and visual textures is known to be important for optimum camouflage performance^31^, but many species are unable to change their appearance in real-time according to the surroundings. Instead, polymorphism often occurs according to the distinct subtypes of the habitat to minimize the detectability of the target against a specific microhabitat^23,49^. Even for the species with active and dynamic camouflage function, the expressible camouflage patterns are not fully arbitrary in many cases but limited to certain modes, e.g., stipple, mottle, and disruptive patterns found in cuttlefish^60^. In this regard, as a compromise suggestion to the pixelated scheme, we propose the artificial camouflage with a controllable polymorphism that enables the display of only selected textures, which appears to be effective for a wide range of camouflage applications that subject to a limited number of habitats.

**Fig. 3:**
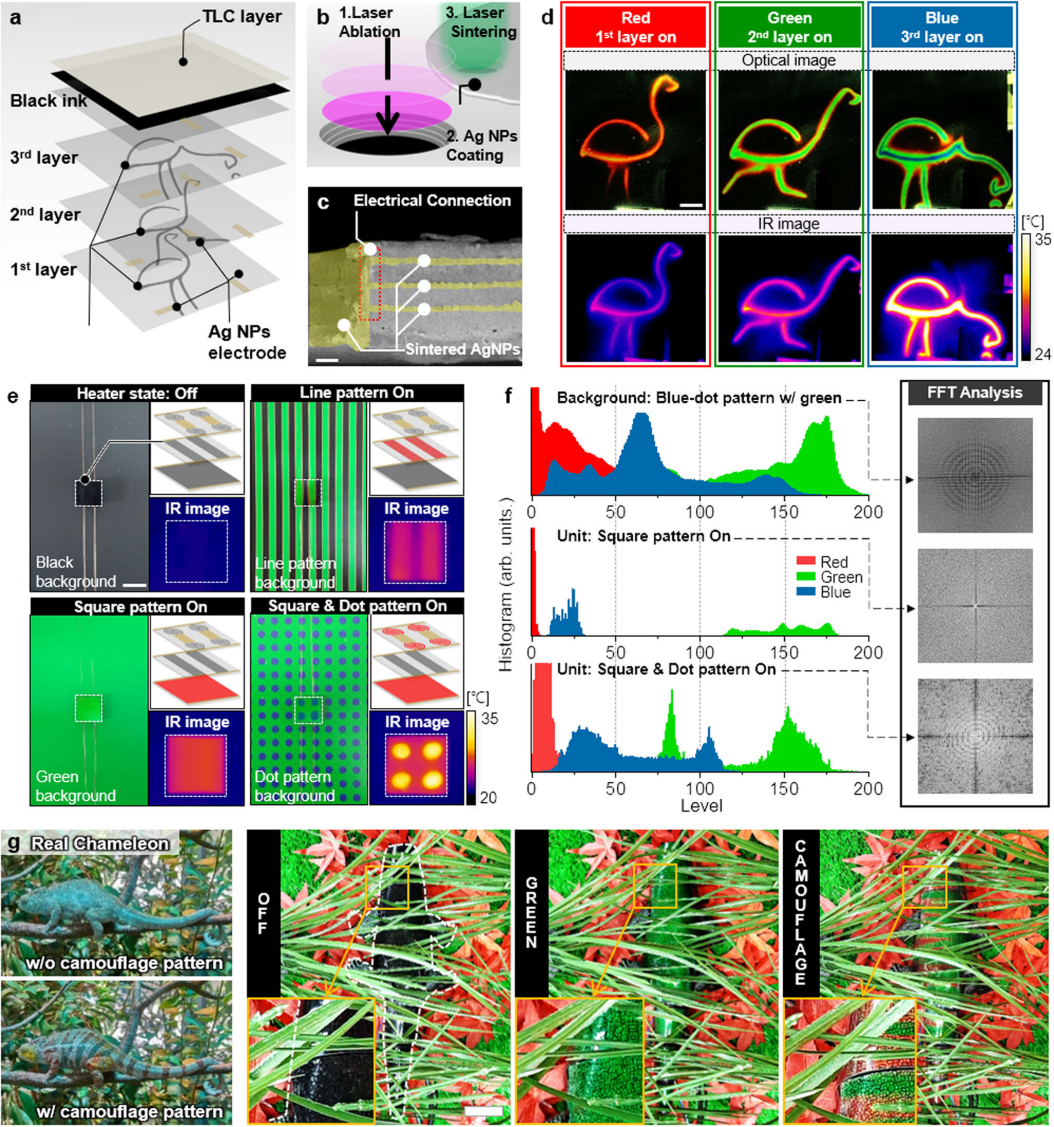
The ATACS with a multi-layered heater for effective camouflage in microhabitat. **a** Schematics of the ATACS with multi-layered 3 ostrich patterns. **b** A laser-assisted fabrication process for stacking heater layer. **c** SEM image of the ATACS’s cross-section. The electrical connection between sintered Ag NPs electrodes is successively formed. The scale bar is 10 µm. **d** Digital and IR image of colorful running ostrich demonstration. The scale bar is 5 mm. **e** Concealment of the multi-layered ATACS from the background by color and pattern matching. Inset IR images show the heater state corresponds to pattern and color. The scale bar is 15 mm. **f** Color and pattern similarity analysis: RGB histogram from digital images of blue-dot pattern with a green background, the ATACS with green square pattern, the ATACS with a green square, and blue-dot pattern. **g** Effectiveness of camouflage pattern for crypsis. Visual comparison of a real chameleon and chameleon model covered with 7 multi-layered ATACS. The scale bar is 5 cm.

The key feature that enables the controllable polymorphism with highly mitigated complexity is the vertically stacked Ag NW heaters. The ATACS with 3 Ag NW heating layers at distinct ostrich patterns, which resembles the snapshots from a moving ostrich (Fig. 3a), is prepared to verify that the temperature profile is generated at each layer can be relocated to the outer TLC layer. To secure large-area compatibility and benign processing environments, laser ablation and laser sintering techniques are employed (Fig. 3b) in ambient conditions to pattern the Ag NW heater into the designated design and to connect the readout electrodes including the ground wire that penetrates through the multi-layered heaters as shown in the cross-sectional SEM image in the Fig. 3c (Supplementary Fig. 18 for the full fabrication steps). Each Ag NW heater, which is operated by a single input voltage, is activated to create distinct ostrich patterns at RGB colors respectively (Fig. 3d), and the corresponding real images together with the IR images confirm that the temperature profile from the individual layer is transferred to the outermost layer independent to the effects from the rest, owing to the ultrathin layer thickness and the large percolation voids of the Ag NW heater. (Supplementary Movie 3 for the real-time repeated activation of ostrich patterns, Supplementary Figs. S15–S18 for the control dependence and layer uniformity with respect to the multi-layered configuration) It is worth mentioning that the corresponding ATACS only exploits 3 input channels in total. Since the smallest feature size involved in this demonstration is at 1 mm regime in 30 mm × 30 mm active area, 900 pixels are expected to display these images at similar resolution by the conventional laterally pixelated scheme, which is only conceivable through highly precise, multi-step fabrication method and extremely complicated control system. Furthermore, in the study to evaluate the resolution and camouflage accuracy of the ATACS, ATACS shows 0.5 mm smallest feature resolution and shows high accuracy comparable with 64 × 64 pixelated camouflage device (Supplementary Figs. 23–27 and Supplementary Table 1). In this regard, the vertically stacked Ag NW heater can be a much more competitive option when the number of target patterns is restricted.

The introduction of artificial microhabitats assists the investigation of the effectiveness of delivering the fine patterns for camouflage performance. Apart from the background color, we assume that the terrain may include two types of microhabitats, i.e., line pattern and dot pattern. In accordance with the given information, the ATACS is prepared to include three distinct heaters with square, line, and dot patterns. The activation of a single heater or a combination of heaters enables not only the matching of the background color (square pattern on) or the pattern (line pattern on) but also simultaneous matching of the background color and the pattern (square and dot pattern on) as shown in Fig. 3e. The degree of matching between the background and the ATACS in terms of the color and the incorporated pattern can be characterized more quantitatively by the histograms of RGB intensities and Fast Fourier Transform (FFT) image. Once the target background is not monotone, e.g., blue-dot pattern with green background as shown in Fig. 3f, it is evident that neither the color nor the pattern can be well-matched by the ATACS with a single square heater, but the simultaneous activation of the square and dot heaters brings the histogram and the FFT image to be a close resemblance to the target background, implying that both of the background color and the visual textures are now analogous.

The importance of fine patterns for more natural camouflage characteristics is observable from the actual species, seeing that the chameleon image after deliberately erasing the fine patterns from the original image becomes more perceptible from the background (Fig. 3g). Likewise, the chameleon model in grass demonstrates that the degree of concealment can be enhanced as the multilayer heater is added to the ATACS. Figure 3g shows three different states of the chameleon model whose surface is covered with seven multi-layered ATACS patches, similar to the one in Fig. 1g. Each camouflage patch includes two Ag NW heaters laser-patterned at uniform and curved-line patterns. By activating the uniform heaters, (middle image) the chameleon model exhibits green color to be more alike to the background compared to the off state, (left image) yet the concealment is not perfectly natural as the whole body displays the same color as a lump. Displaying distinct but uniform colors on each pixel (e.g., alternating red and green pixels) is also not very appealing in improving the concealment since there are only a few pixels with clear boundaries. On the other hand, the surface created by activating two heaters at the same time as shown in the right image of Fig. 3g demonstrates that the camouflage performance can be enhanced through our strategy as the curved-line pattern mimics the texture of the surrounding grass to achieve more natural blending to the ambient.

### Instantaneous camouflage

**Fig. 4:**
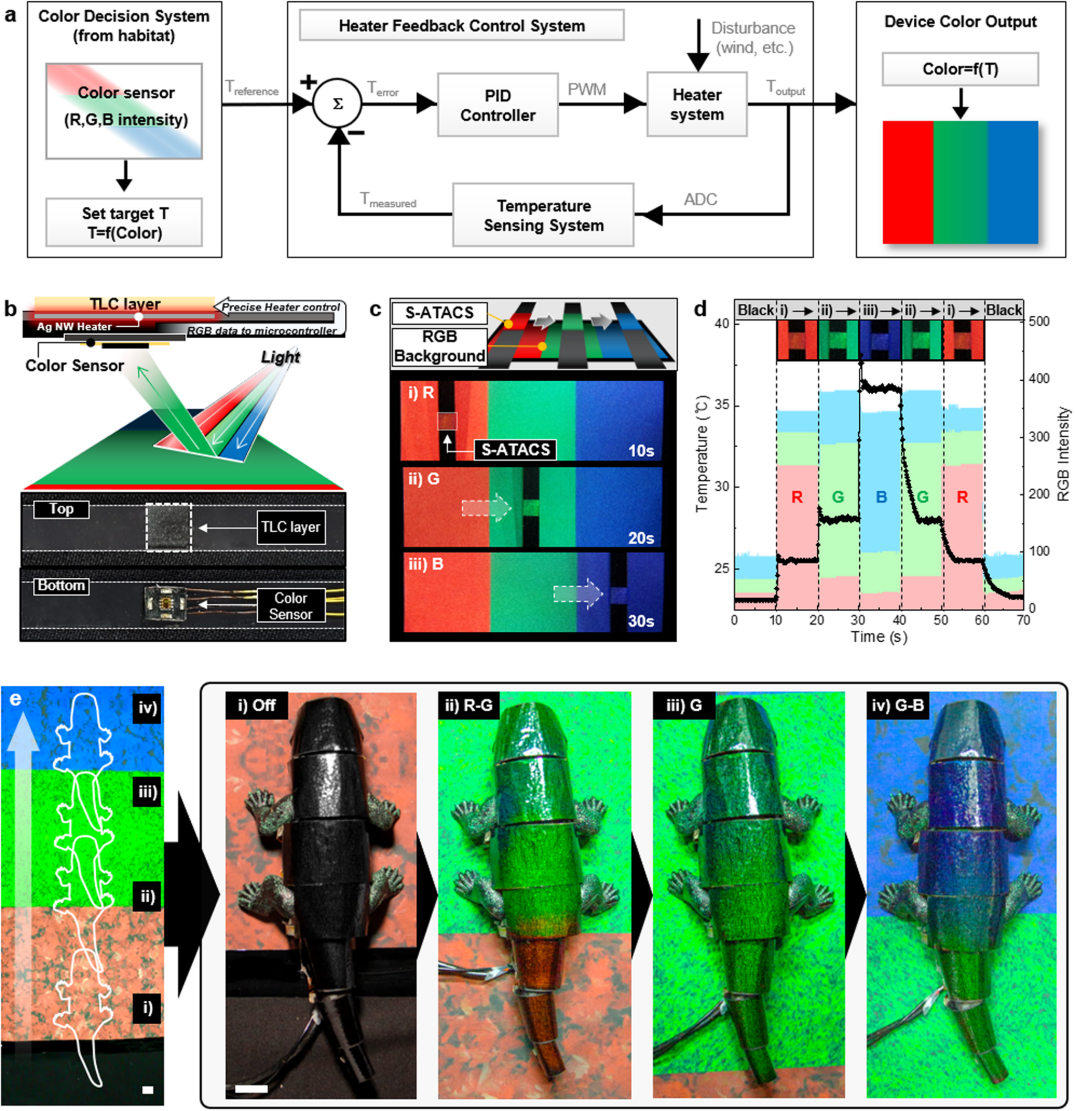
Demonstration of autonomous S-ATACS with instantaneous crypsis ability. **a** Schematics of autonomous camouflage system with heater feedback control logic circuit. **b** The ATACS with color sensor and its schematics of working principle. **c** Demonstration of S-ATACS that changes its color using real-time background color data. **d** The temperature of the ATACS and RGB intensity of real-time color sensing data from the background. Insets are the digital image of S-ATACS color changes with respect to RGB intensity. **e** Instantaneous crypsis of the chameleon robot. The robot crawls on the background and changes its skin color according to the background color. The scale bar is 3 cm.

The camouflage-generation strategy in the actual living species is hypothesized to be composed of several steps including the collection of visual information through the retina and recurrent circuits in the brain for skin patterning control through motor neurons^61^. Likewise, the artificial camouflage system should be operated autonomously by covering the entire process of background color retrieval and the delivery of matching surface color. A direct and simple model is implemented for the development of an autonomous Sensor-integrated ATACS (S-ATACS). Instead of analyzing the visual information from high-density arrays, the local color for each pixel is directly acquired from the RGB intensity measured from a color sensor to set the target temperature. The target temperature is then reached and maintained by the PID controller to match the local background color (Fig. 4a). Since the resultant system operates locally and independently, the complete version of the device can be established by the simple assembly of the individual units. The configuration of the S-ATACS is schematically illustrated in Fig. 4b together with the optical photographs of the top and bottom sides of the S-ATACS. Since the sensing unit and the ATACS are not required to be in contact with each other, the target body, as well as the control circuit, can be inserted between these two-component to be concealed by displaying the background color at the outermost surface. The autonomous camouflage function of the S-ATACS is verifiable from the optical images and the temperature evolution according to the underlying RGB intensities as shown in Fig. 4c, d. As the autonomous S-ATACS moves to the region with different background colors, the TLC color changes promptly according to the measured RGB intensities.

As a final demonstration of the proposed artificial camouflage strategy, an autonomous and adaptive S-ATACS is applied to the moving chameleon robot (Supplementary Fig. 29). The developed S-ATACS are attached conformally to the exterior of the chameleon and a case scenario is set as shown in Fig. 4e. The artificial camouflage is activated as the chameleon model moves across 3 different habitats exhibiting RGB colors, denoted as R-, G- and B-habitat, respectively. The images are the snapshots taken while the chameleon model is at R-G habitats (Fig. 4e, ii), G habitat (Fig. 4e, iii), and G-B habitats (Fig. 4e, iv) demonstrating significantly improved blending into the surroundings compared to the deactivated chameleon model (Fig. 4e, i). The real-time operation of the proposed camouflage device under the moving chameleon model can be observed in Supplementary Movie 4. Fully automated color matching in accordance with the change in the local background, together with the exceptionally natural and rapid transition characteristics, verifies that our strategy possesses great potential as a next-generation artificial camouflage.

## Discussion

Artificial camouflage is an important technology for the military, and the relevant market is growing rapidly at the moment^62^. Recent developments on smart textiles for the military purpose also increases the interest towards adaptive camouflage technologies^63^. Besides military applications, artificial camouflage receives broad attention from architecture^64^, art and fashion^65^, and a number of consumer products for hunting and outdoor activities^66^.

The TLC and multiple Ag NW heaters, combined in a vertical structure, have been presented as a novel system with high potential for artificial camouflage purposes. The issues raised from the TLC layer, which are also probable in other thermochromic camouflage schemes, are largely resolved by the adaptation of an active feedback control system based on the stable and comparatively high TCR of the Ag NW heater. The intrinsic mechanical and electrical stability of the Ag NW percolation network further enables its operation during continuous mechanical bending and external environmental changes, which are anticipated for most camouflage applications. The outstanding coloration and transition characteristics, together with the fully automated matching to the local background color through color sensing and active control, significantly improves the blending of the target body to the surroundings, presenting the great potential of the proposed scheme for highly effective, next-level artificial camouflage devices.

One of the distinctive and motivating topics brought in this study is the novel method to express the fine patterns through the superposition of the temperature profiles from the vertically integrated Ag NW heaters. The corresponding scheme is proposed to improve the camouflage characteristics at the minimum expense of the system complexity through an entirely separate route. For the optimum operation, the slow-varying background color is matched by the coarse lateral pixels with the uniform heater, while the detailed patterns, predefined according to the representative microhabitats, are superimposed by the vertically stacked heaters based on the perceived patterns. However, the activation of the secondary heaters, which can be regarded as an optional function for more advanced camouflage performance, has been excluded from the final demonstration of the moving chameleon, due to the absence of an appropriate pattern recognition algorithm at the current stage. For the efficient recognition and expression of the high-resolution surface texture, the visual information has to be processed in advance to regulate the input signals for the set of multilayer heaters. Since the given problem is expected to be dependent on the multidimensional parameters such as the moving velocity, the number of the heaters and the selected patterns, the number and the arrangement of sensing units as well as the characteristics of human perception, we expect that the active cooperation with professionals of diverse backgrounds, including signal processing and data-driven science, is necessary to facilitate further progress in the high-performance artificial camouflage research for higher impact.

## Methods

### Ag nanowire synthesis

Modified polyol and a one-pot process were used to synthesis long Ag NW (≥100 µm in length, ≤100 nm in diameter). 0.4 g of PVP (*M*_w_ ≈ 360 000; Sigma-Aldrich) and 0.5 g of silver nitrate (AgNO3, Sigma-Aldrich) are sequentially dissolved in 50 mL of ethylene glycol (EG, 99.9%; Sigma-Aldrich) by a magnetic stirrer. Then, 800 µL of CuCl2·2H2O solution (3.3 × 10^−3^ m) was rapidly injected into the mixture and stirred mildly. The mixture solution was suspended in a preheated silicone oil bath at 150 °C. The growth of Ag NWs was maintained for 3 h. After the growth is finished, the mixture solution is separated using acetone first and cleaned by using centrifugation of 1257.57 × *g* for 100 min with ethanol (EtOH, 99.5%; Samchun Chemicals). This cleaning process is repeated at least three to four times to securely remove the organic residue such as PVP on the surface of Ag NWs. The cleaned Ag NWs were re-dispersed in EtOH.

### Ag nanoparticle synthesis

Modified polyol method was used to synthesize silver nanoparticles (Ag NPs). As a precursor, silver nitrate (0.25 mol/L, 99%; Sigma-Aldrich) was dissolved in ethylene glycol (EG, 99.9%; Sigma-Aldrich) with 0.02 mol/L of polyvinylpyrrolidone (PVP, *M*_w_ ~10,000; Sigma-Aldrich). The mixed solution was stirred at 150 °C and 3.14 × *g* magnetic stirring until the synthesis was completed. Then, the synthesized Ag NPs were centrifuged at 6847.75 × *g* for 30 min and cleaned by EtOH. The purified Ag NPs were re-dispersed in EtOH for the use

### Preparation of multi-layered ATACS

All fabrication processes consist of repeated procedures: polymer annealing, fabrication of patterned silver nanowire heaters on each layer, and silver nanoparticle electrodes for the electrical connection between the layers. The shape of the heater and the number of stacked layers could be easily modified according to the process so that ATACS fabrication with various multi-patterns is possible. First, cPI varnish (Colorless Polyimide Varnish, IPITECH), as a substrate, is spin-coated on glass (559 × *g*, 1 min), and annealed with the increasing temperature gradually to 300 °C on the hot plate. Ag NPs inks are coated on the annealed cPI film using a spin coater (5.59 × *g*, 1 min), and then sintered by a selective laser process to form a base electrode. Unsintered Ag NPs are washed with ethanol. On this base electrode, the Ag NWs solution is spray-coated and then patterned by a laser ablation process to complete the heater with a designed pattern. As a separate layer of stacked heaters, while protecting the heater electrode, the cPI is coated on the Ag NW heater by the same procedure as the first cPI layer. After that, holes are laser-ablated on the target area to form via where the Ag NPs electrode of each layer and the external electrode are connected. The Ag NPs inks are spin-coated and selectively sintered using a laser process to form an electrical connection through the via. These processes are repeated twice to form a heater in which three different patterns are stacked. Finally, a multi-layered ATACS is fabricated by sequentially spin coating black ink (acrylic inks, Culture Hustle) and TLC ink (Sprayable liquid crystal ink, SFXC) on the top of stacked heater regions. Schematics of the whole fabrication process are depicted in Supplementary Fig. 18.

### Implementation of the electronic system

The microcontroller (Arduino Mega 2560, Arduino) determines target color based on the overall brightness and dominancy between red, green, and blue lights measured by a color sensor (TCS3472, AMS). The color sensor provides its data through Inter-Integrated Circuit (I2C), a bus-type serial communication protocol, hence the microcontroller can request multiple sensors their data in sequence through a common communication channel, easing the complexity of the circuit wiring. The output data used in this study are ranged from 0 to 255 in proportion to the RGB intensity, and the sampling rate is 10 Hz for each color sensor. Estimation and control of heater temperature are conducted at 30 Hz. To enhance the control precision, the change in electric resistance (~Δ*T*) of the heater is magnified with an operational amplifier (LM324, Texas Instruments), and high-frequency noise is suppressed by a low-pass filter implemented in software with a cutoff frequency of 300 Hz.

### Characterizations

To analyze the effective camouflage ability of ATACS, we conducted a basic analysis focusing on the Fast Furrier Transform image and color histogram comparison between the ATACS and its background. We first photograph digital images of a green background with a blue-dot pattern, unit of green square pattern, and unit of blue-dot pattern with green square pattern (Fig. 3e). Then, we plot the RGB histograms represented by an 8-bit number between 0 (dark) and 255 (bright) using Origin (OriginLab®) to compare color similarity between each circumstance (graphs in Fig. 3f). The *y* axis value represents the amount of data with respect to the brightness value of the *x* axis. Also, to pattern similarity analysis, we convert each image to grayscale and then transform it into an FFT image using Origin (OriginLab®). The FFT image shows a spatial frequency spectrum using a two-dimensional Fourier transform to follow the image in the uniaxial direction (*x* or *y* axis). From the converted FFT image, we could figure out the pattern similarity of each sample, visually and intuitively.

## Supplementary information

Supplementary Information

Description of Additional Supplementary Files

Supplementary Movie 1

Supplementary Movie 2

Supplementary Movie 3

Supplementary Movie 4

## Data Availability

The authors declare that all data supporting the findings of this study are available within the main text and Supplementary Information. Source data for the figures are available at 10.6084/m9.figshare.14853753.v1.
