# Supplementary material for: Biomimetic chameleon soft robot with artificial crypsis and disruptive coloration skin
Source: Nat Commun. 2021 Aug 10;12:4658. doi: 10.1038/s41467-021-24916-w (PMC8355336; doi:10.1038/s41467-021-24916-w)
Supplement: Supplementary file 1 — Supplementary Information [file 41467_2021_24916_MOESM1_ESM.pdf]

Supplementary Information for

Biomimetic Chameleon Soft Robot with Artificial Crypsis and Disruptive Coloration Skin

Hyeonseok Kim[1,†], Joonhwa Choi[1,†], Kyun Kyu Kim[1], Philip Won[1], Sukjoon Hong[2,*], Seung Hwan Ko[1,3,*]

[1] Applied Nano and Thermal Science Lab, Department of Mechanical Engineering, Seoul National University, 1 Gwanak-ro, Gwanak-gu, Seoul, 08826, Korea

[2] Optical Nanoprocessing Lab, Department of Mechanical Engineering, BK21 FOUR ERICA-ACE Center, Hanyang University, 55 Hanyangdaehak-ro, Sangnok-gu, Ansan Gyeonggi-do 15588, Korea

[3] Institute of Advanced Machinery and Design (SNU-IAMD)/Institute of Engineering Research, Seoul National University, Gwanak-ro, Gwanak-gu, Seoul 08826, Korea

This Supplementary Information includes:

Supplementary Note 1. Thermochromic liquid crystal (TLC)
Supplementary Note 2. The performance evaluation of the ATACS under the various surrounding temperature
Supplementary Note 3. The performance of the ATACS under the various surrounding environments
Supplementary Note 4. Mechanical and chemical stability of the ATACS
Supplementary Note 5. Theoretical study and experiment for the evaluation of the multi-layered ATACS' thermal performance
Supplementary Note 6. Camouflage accuracy of the ATACS
Supplementary Note 7. The critical dimension of AgNW patterning
Supplementary Fig. 1. Schematics of ATACS's electrode layering structure
Supplementary Fig. 2. Schematics of the fabrication process of the ATACS
Supplementary Fig. 3. Continuous spectrum graph of ATACS with applied voltage
Supplementary Fig. 4. Stability test: HSV graph of the ATACS during the repeated on-off cyclic test
Supplementary Fig. 5. Temperature response profile of the ATACS with active control at various ambient temperatures
Supplementary Fig. 6. PID control-based feedback system for instantaneous and stable coloration control
Supplementary Fig. 7. Temperature coefficient of resistance (TCR) of the Ag NW heater
Supplementary Fig. 8. The temperature profile of ATACS without feedback control

Supplementary Fig. 9. the ATACS control ability in a relatively high-temperature environment
Supplementary Fig. 10. Optical images of RGB coloration of the ATACS in the water
Supplementary Fig. 11. Performance of the ATACS under the extremely low-temperature
Supplementary Fig. 12. Peeling test of AgNW heater
Supplementary Fig. 13. Chemical stability test of the AgNWs heater under the H_2O_2 condition
Supplementary Fig. 14. The results of the 80-80 test on the AgNW-cPI and cPI-agNW-cPI composite
Supplementary Fig. 15. Mechanical stability test of the ATACS
Supplementary Fig. 16. Long-term durability test of ATACS heater electrode
Supplementary Fig. 17. Electrical resistance sensitivity of ATACS with various AgNW solution densities
Supplementary Fig. 18. Schematics of the multi-layered ATACS fabrication process
Supplementary Fig. 19. Schematics of the AgNW heater configuration with ideal maximum thermal conductivity
Supplementary Fig. 20. SEM image of AgNW-cPI layer to calculate the areal ratio of AgNW
Supplementary Fig. 21. Comparison of thermal transparency between conventional bulk metal stacked heaters and nanowire stacked heaters
Supplementary Fig. 22. Numerical simulation to evaluate how the number of the heater layer affects the performance of the temperature control
Supplementary Fig. 23. The on-off cyclic test of the silver nanowire heater
Supplementary Fig. 24. Pattern accuracy evaluation
Supplementary Fig. 25. Schematics of N x N heater array circuit
Supplementary Fig. 26. ATACS's feature resolution evaluation
Supplementary Fig. 27. The critical dimension of AgNW patterning
Supplementary Fig. 28. Color and pattern of the ATACS similarity analysis
Supplementary Fig. 29. Schematics of ATACS attached on the chameleon robot body
Supplementary Fig. 30. Specific dimensions of the heater patterns of ATACS on the chameleon model
Supplementary Table 1. Previous studies on artificial camouflage device that incorporates pixelated scheme to created spatially varying patterns

Supplementary Note 1. Thermochromic liquid crystal (TLC)

The liquid crystal can be classified on the basis of their different structures and properties, and the specific TLC used in this study belongs to the chiral nematic (cholesteric) liquid crystal. The structure of the cholesteric mesophase consists of sheets of molecules that behave as in the nematic phase, while each nematic director axis is skewed at some displacement angle with respect to the adjacent sheets. (Inset image of the Fig.1b) The longitudinal distance required for the nematic director axis to rotate one complete revolution is called the pitch length (p). Chiral nematic liquid crystal, therefore, forms a helical structure on a larger scale. and several interesting optical properties are introduced due to the internal structure. For each layer, two refractive indices can be defined, either parallel to the director or at right angles to the director. As a consequence, the TLC layer exhibits circular dichroism when a plane-polarized beam of light enters the helical structure that has the same pitch length as the incident wavelength: one circularly polarized beam is transmitted while the other component is reflected. The wavelength subject to the circular dichroism can be expressed as $\lambda = \bar{n}p$, where $\bar{n}$ corresponds to the average refractive index.[67] When an ordinary white light is entered instead of a single wavelength, each wavelength will experience a different refractive index to result in a reflective color band.[68] Meanwhile, the pitch (p) and hence the wavelength of the reflected band decrease with increasing temperature for almost all chiral nematic compounds.[57] As a result, the reflective color from the TLC layer shows red at low temperature and blue-shifted at high temperature as shown in our demonstration.

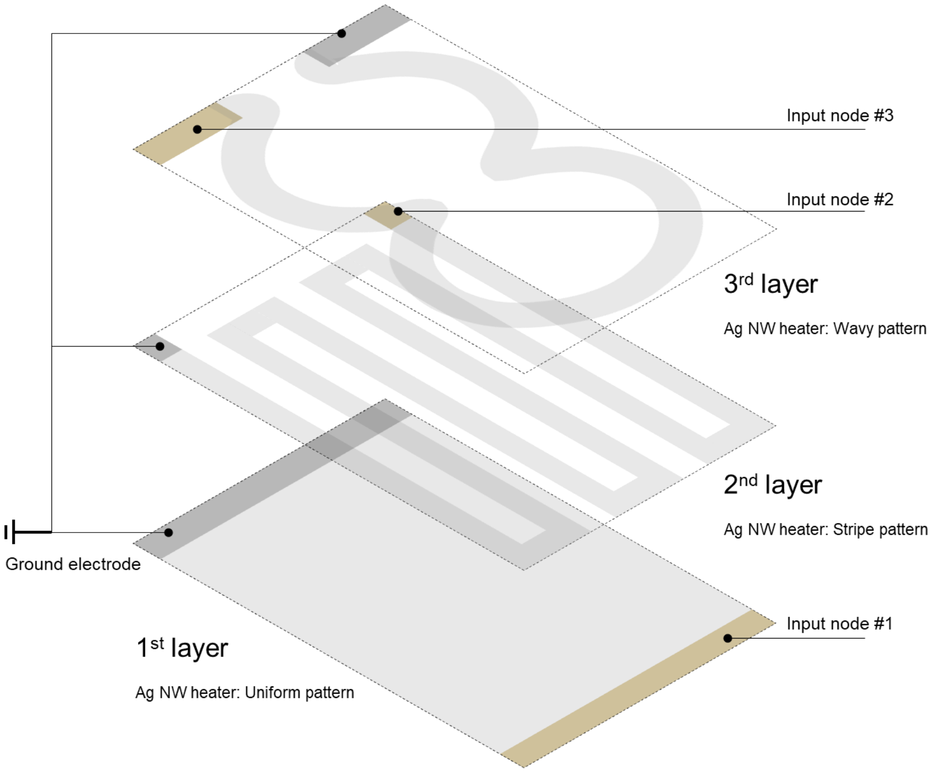

Supplementary Fig. 1. Schematics of ATACS's electrode layering structure. The ground electrode of each layer is connected vertically by via, and each input node is connected to the external electrode separately.

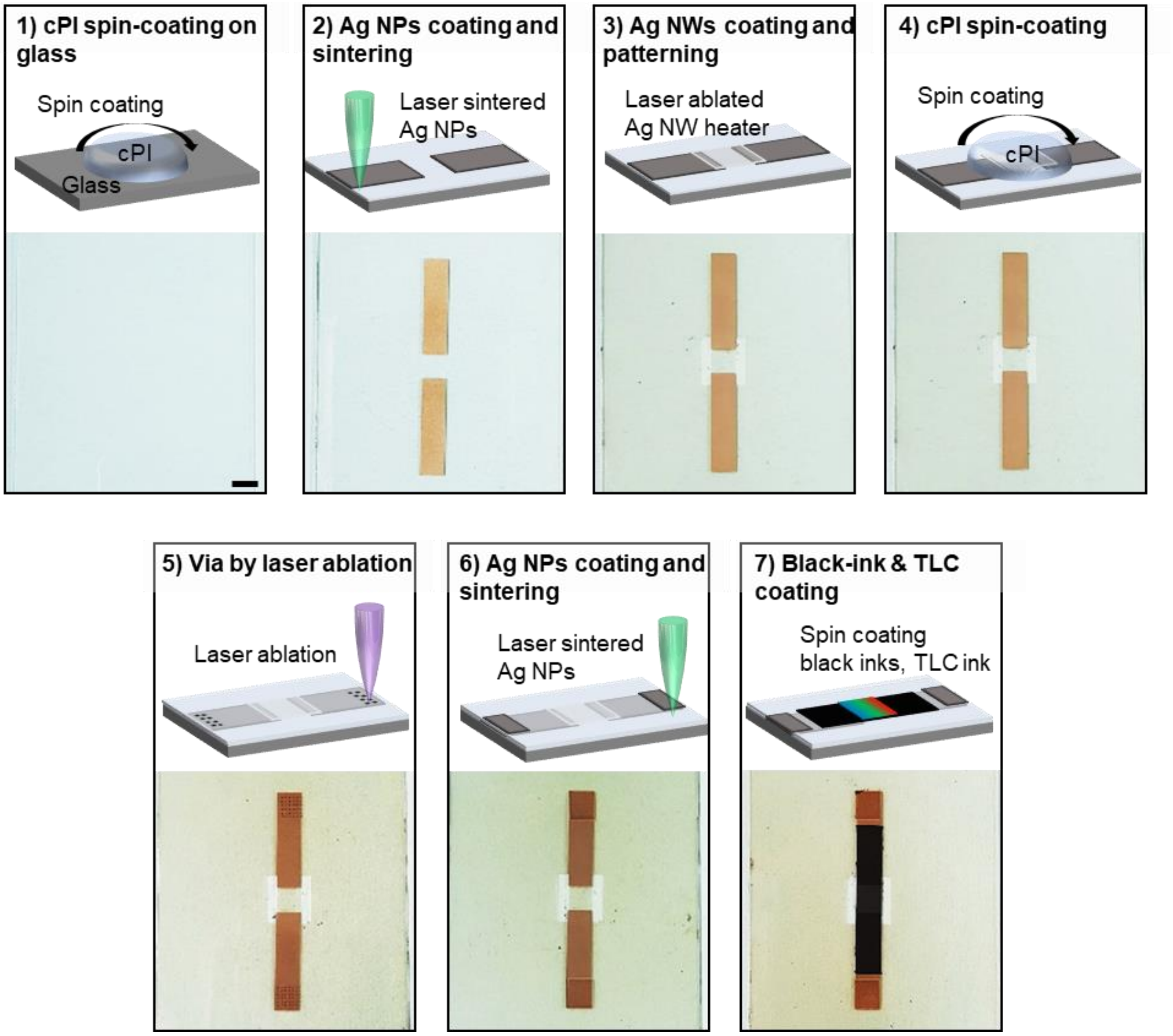

Supplementary Fig. 2. Schematics of the fabrication process of the ATACS. 1) cPI varnish is coated on the glass substrate using spin coater and then coated cPI is annealed with gradually increasing temperature to 300 ^{o}C. The scale bar is 5 mm. 2) Silver nanoparticles (Ag NPs) ink is coated on the cPI layer using a spin coater and selectively sintered by a laser process. 3) For fabricating an Ag NW heater between two Ag NP electrodes, Spray-coated Ag NWs are processed by laser ablation. 4) cPI is coated and annealed on the Ag NW heater as a cover layer. 5) Drilling of cPI surface by laser ablation process. 6) A electrical connection between the Ag NP electrode

and the top layer is fabricated by coating Ag NPs ink and laser sintering process sequentially. 7) Spin coating black ink and TLC ink sequentially on the Ag NW heater area.

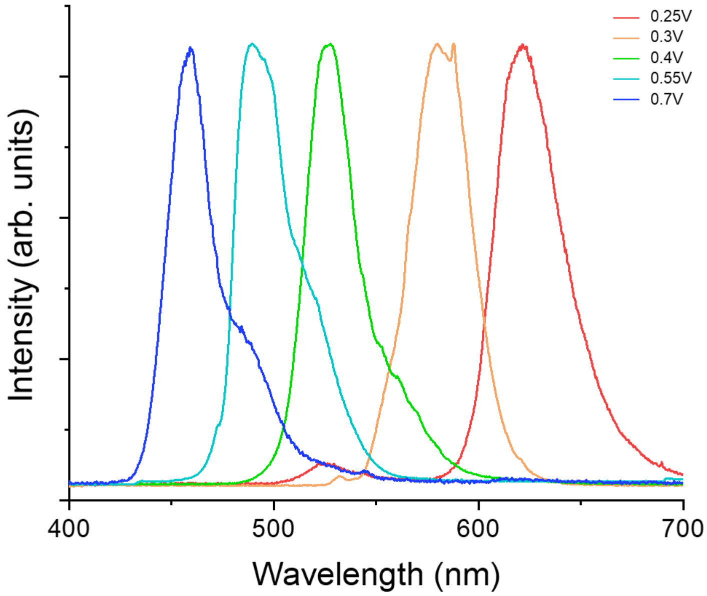

Supplementary Fig. 3. Continuous spectrum graph of ATACS with applied voltage. ATACS shows wavelength peaks 459 nm for 0.25 V, 490 nm for 0.3 V, 528 nm for 0.4 V, 580 nm for 0.55 V, and 621 nm for 0.7 V.

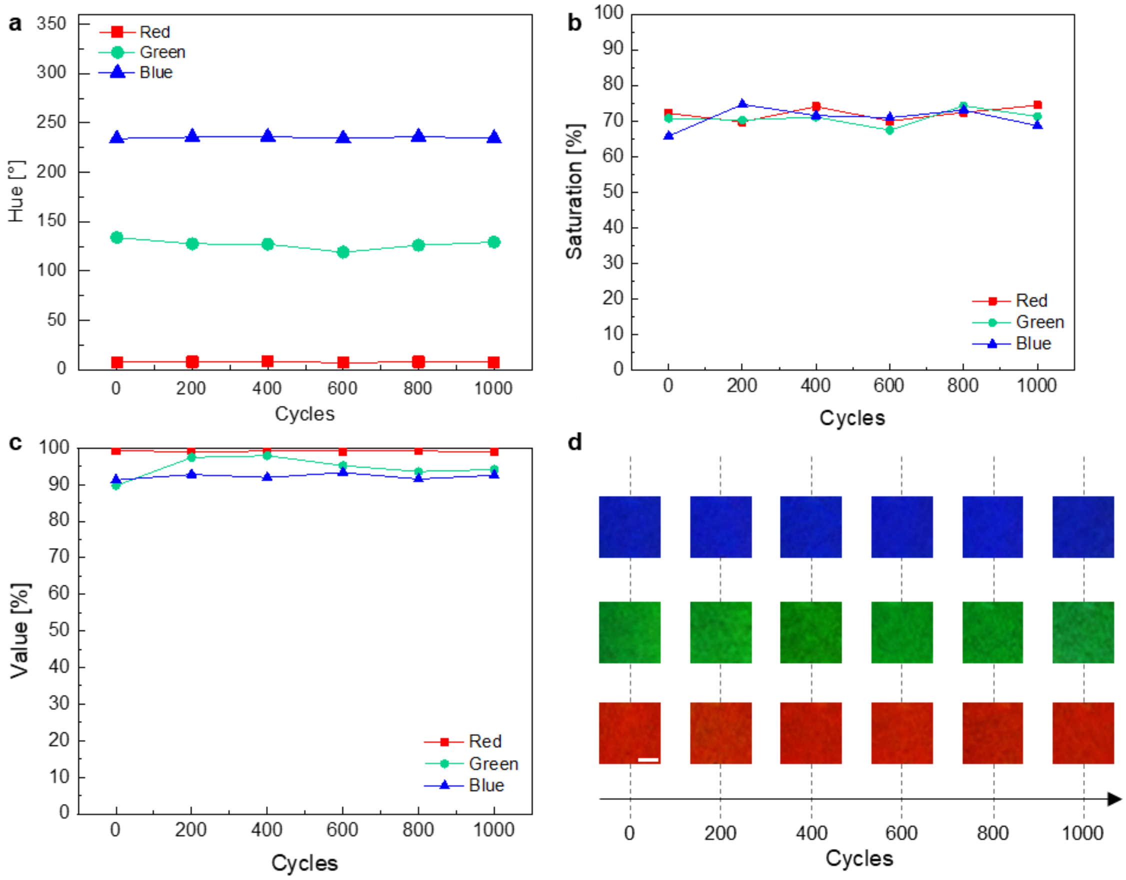

Supplementary Fig. 4. An on-off cyclic test to evaluate the stability of the ATACS. Each graph indicates a hue **a**, saturation **b**, and value **c** of the ATACS during 1,000 cycles on-off test and shows consistent and stable color performance even under high repetition conditions. **d** Actual image of the ATACS in each cycle. The ATACS shows great repeatability of color generation during 1000 cycles. The scale bar is 2 mm.

Supplementary Note 2. The performance evaluation of the ATACS under the various surrounding temperature

Evaluating the performance of ATACS in a room temperature environment as well as in a variety of temperature drop environments is valuable information for appraising its capabilities and versatility. In order to measure the response time in various temperature drop environments, an additional heater control study of ATACS was conducted at an ambient temperature of 15 °C, 5 °C, -5 °C. We set the target temperature of the ATACS to 25.5 °C for the red color, 28 °C for green, and 36 °C for blue. The ATACS changes color to R, G, B with active control at each ambient temperature, and the temperature profile is shown in Supplementary Fig. 5. ATACS took 0.51s to reach the red color at -5 °C, 0.49s at 5 °C, 0.46s at 15 °C, and took 0.57s to reach the blue color at -5 °C, 0.49s at 5 °C, 0.48s at 15 °C. The lower the ambient temperature and the higher the target temperature, the characteristic time becomes longer because a larger temperature rise is required to reach the target temperature.

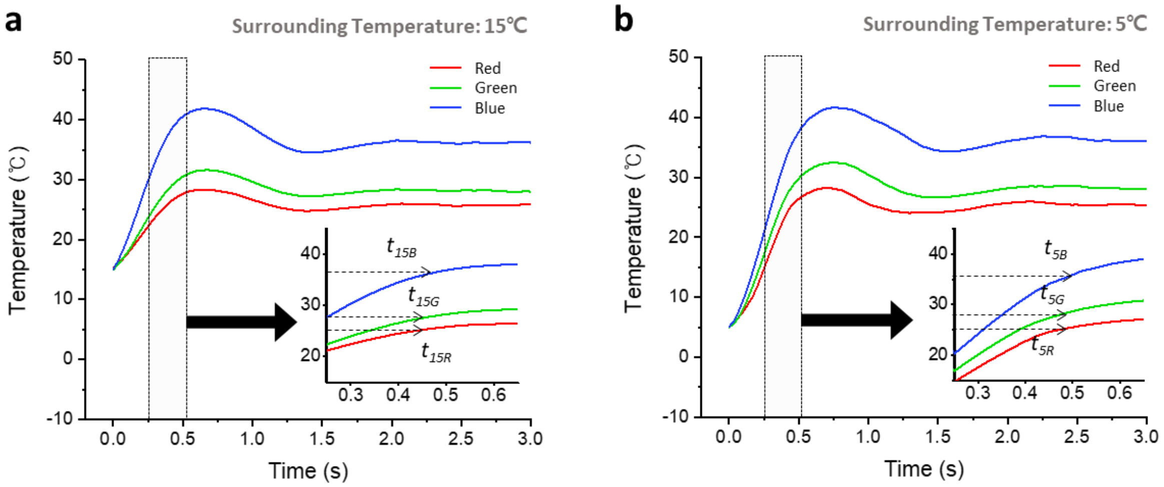

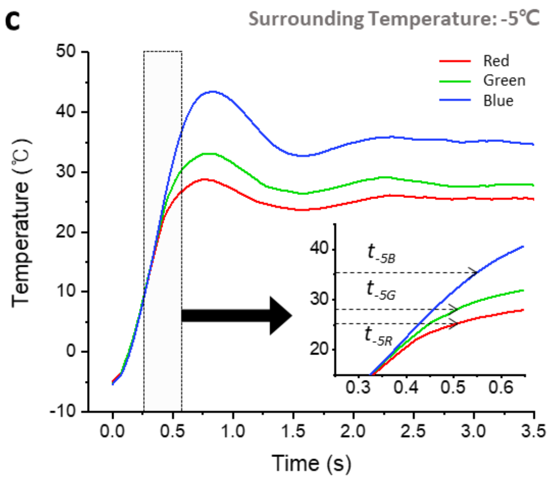

d

Temp. (℃)	Target Color	Characteristic Time (s)
15	R	0.46
	G	0.48
	B	0.48
5	R	0.49
	G	0.49
	B	0.50
-5	R	0.51
	G	0.51
	B	0.57

Supplementary Fig. 5. Temperature response profile of the ATACS with active control at various ambient temperatures. a 15 °C, **b** 5 °C, **c** -5 °C. **d** The characteristic time to target color at each surrounding temperature.

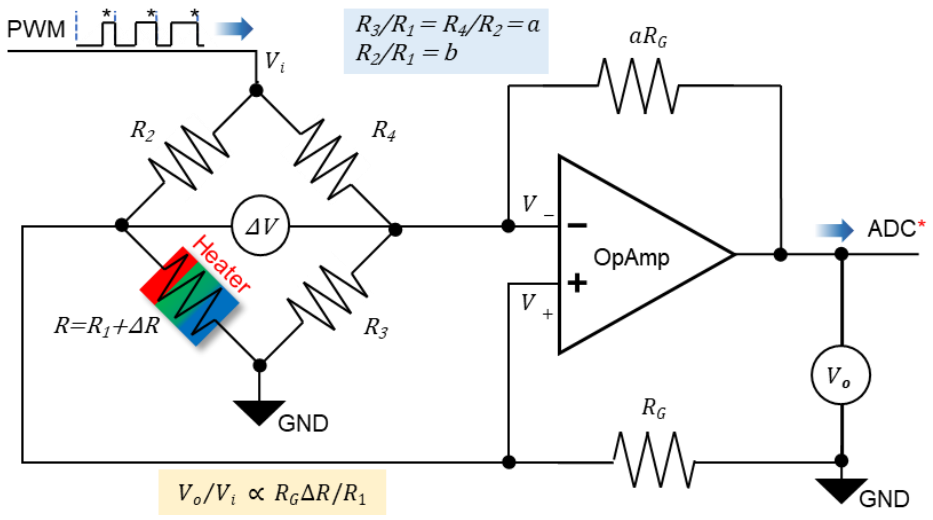

Supplementary Fig. 6. PID control-based feedback system for instantaneous and stable coloration control. The electric circuit of the heater feedback control system. The power of the heater linearly increases with the duty ratio of the PWM generated by a microcontroller. The conventional Wheatstone Bridge amplifier circuit for $\Delta R/R_1$ measurement is modified to lower the overall power consumption in the system while concentrating most of the power on the heater. The ADC operates (*) only when the PWM signal is in a high state.

Calculation of the gain V_0/V_i of the heater temperature sensing circuit in Supplementary Fig. 6 by Kirchhoff's current law,

$$\frac{V_i - V_+}{R_2} + \frac{V_{GND} - V_+}{R} + \frac{V_{GND} - V_+}{R_G} = 0$$

$$\frac{V_i - V_-}{R_4} + \frac{V_{GND} - V_-}{R_3} + \frac{V_{GND} - V_+}{aR_G} = 0$$

Here,

$$V_{GND} = 0,\ \ V_- = V_+$$

$$R_3/R_1 = R_4/R_2 = a$$

$$R_2/R_1 = b$$

Applying the conditions,

$$V_0/V_i \;=\frac{R_G}{R_2}\left(\frac{1/R_G+1/R_1+1/R_2}{1/R_G+1/R+1/R_2}-1\right)$$

$$1/R_1=\frac{1}{R(1-\Delta R/R)}\;\approx\frac{1}{R_1}(1+\Delta R/R_1)\;\text{ for } \Delta R/R\ll 1$$

Therefore,

$$V_0/V_i\approx\frac{R_G}{R_2}\left(\frac{\Delta R/{R_1}^2}{1/R_G+1/R_1+1/R_2}\right)$$

Typically, $R_G \gg R_1\; and\; R_2$. hence,

$$V_0/V_i\approx\frac{R_G}{R_1R_2(1+R_1/R_2)}\Delta R$$

$$=\frac{R_G/R_2}{(1+1/b)}\frac{\Delta R}{R1}$$

The overall power consumption P in this circuit is

$$P\approx\frac{1+1/a}{1+b}\;\frac{{V_i}^2}{R_1}$$

Although ordinary Wheatstone bridge amplifiers set $a=b=1$, this combination causes inefficiency in power consumption i.e., not only the heater but also the other resistors consume much electric energy. To minimize the waste of energy, the heater system is tuned to have $a=1{,}000$ and $b=4$.

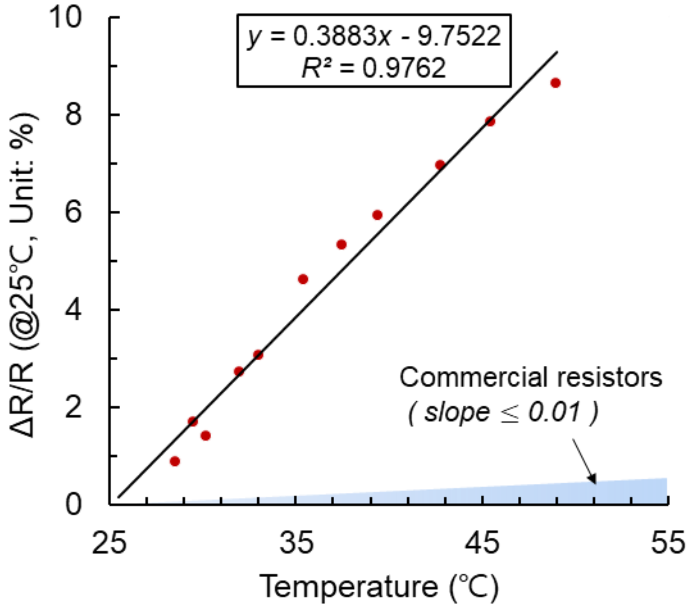

Supplementary Fig. 7. Temperature coefficient of resistance (TCR) of the Ag NW heater. Heater resistance versus temperature. The temperature coefficient of resistance (TCR) of the Ag NW heater is 3.883×10^3 ppm/^{o}C, almost the same as that of the bulk silver. The TCR of commercial high-precision resistors used in the circuit is typically 50~100 ppm/^{o}C.

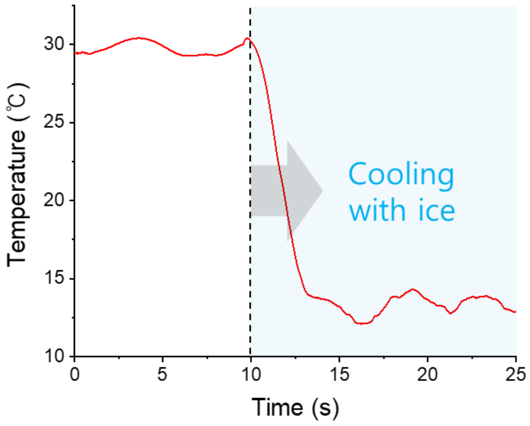

Supplementary Fig. 8. The temperature profile of ATACS without feedback control. A constant DC voltage was supplied to the ATACS to maintain a green state temperature range of 28 to 30 °C. The ATACS was cooled with ice after 10 s causing a surface temperature drops to 15 degrees for 3 seconds. (lower than 15 °C) It is 3 times more a temperature drops than when the feedback control was applied. (Fig. 2b)

Supplementary Note 3. The performance of the ATACS under the various surrounding environments

The ATACS is difficult to drive at high temperatures due to the absence of a cooling device, but it is possible to control the temperature rise at an appropriate level through feedback control. To confirm the ATACS control ability in a relatively high-temperature environment, we measured and plotted the surface temperature and corresponding input power profile where the lighter was turned on and off at a distance of 10 cm from the ATACS and we set the temperature range 28 °C to 31 °C for the green color state. (Supplementary Fig. 9) When the lighter is turned on in a green state, the temperature rises, but at the same time, the input power decreases. As ATACS maintains 31 °C, the input power is also maintained, preventing excessive temperature rise. (Supplementary Fig. 9a) On the other hand, when the feedback control is not applied, the green color of ATACS could not be maintained because a constant input power was maintained and the surface temperature increased by lighter at the same time. (Supplementary Fig. 9b)

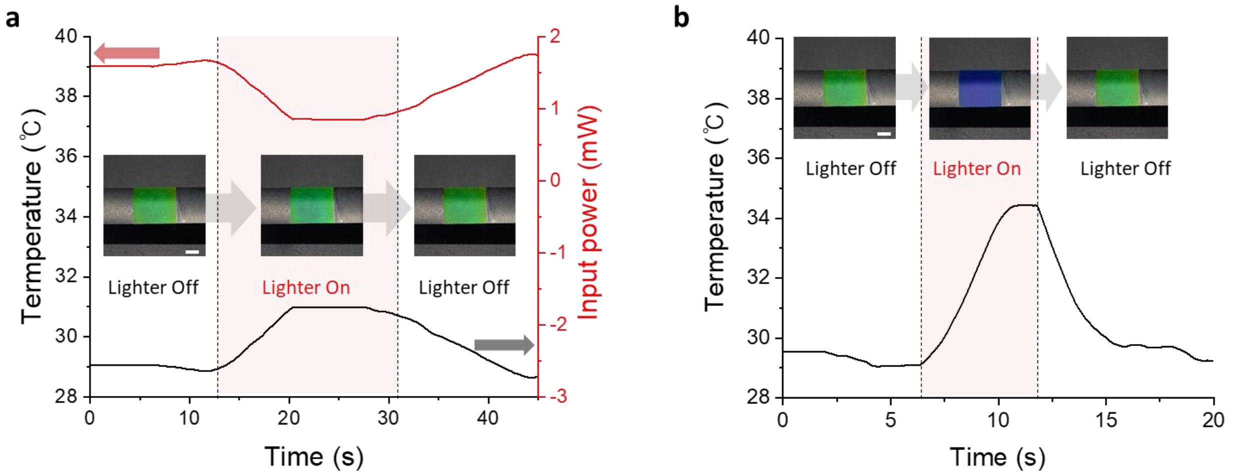

Supplementary Fig. 9. The ATACS control ability in a relatively high-temperature environment a Temperature and input power profile of the ATACS with a feedback control system

under heating with lighter. Inset images are the color state of ATACS with respect to time. The scale bar is 2 mm. **b** Temperature profile of the ATACS without a feedback control system under heating with a lighter. Inset images are the color state of ATACS with respect to time. The scale bar is 2 mm.

Also, verifying that the ATACS can be operated in water is one of the important indicators of the device's performance in extreme environments. Therefore, as an additional experiment to confirm the performance in extreme environments, ATACS was run in water at room temperature. ATACS could show red, green, and blue colors in the water, and it was possible to operate stably in water. (Supplementary Fig. 10)

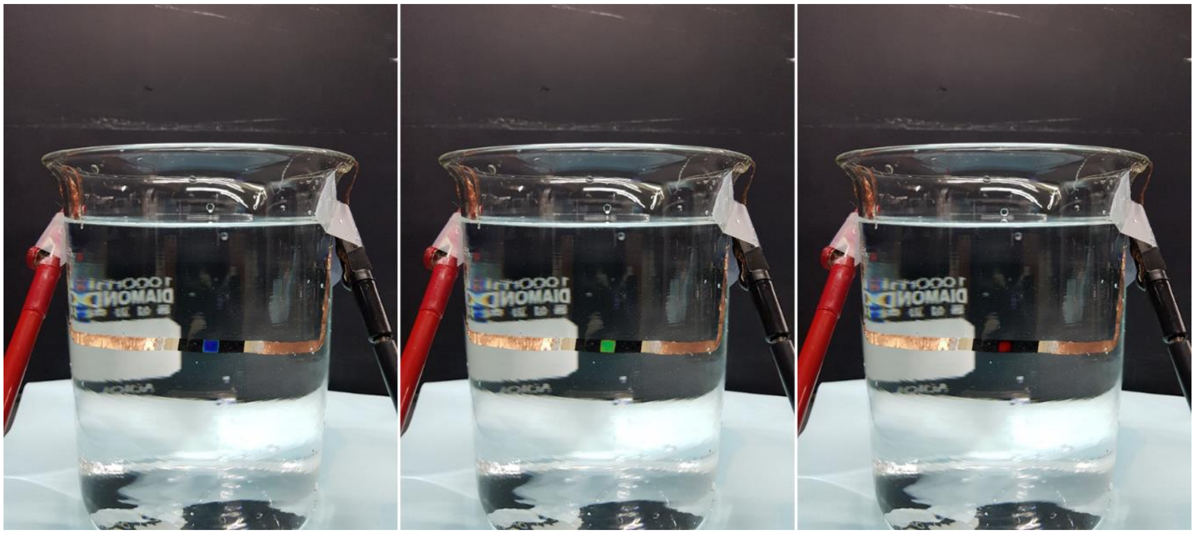

Supplementary Fig. 10. Optical images of RGB coloration of the ATACS in the water.

The heater temperature is proved to be controllable even at -20 ˚C condition as shown in Supplementary Fig. 11a. (characteristic time is 0.55 s, 0.55 s, and 0.63 s for RGB signals respectively) However, it does not mean the coloration of the ATACS has the same performance at the moderate temperature range because the heater temperature measured here does not reflect the overall temperature distribution on the ATACS. Technically, the heater temperature is

measured by reading the overall electric resistance of the heater, hence any information on the resistance distribution over the heater is not available with the current system configuration. Consequently, the ATACS showed non-uniform color distribution attributed to a steep temperature gradient as displayed in Supplementary Fig. 11b.

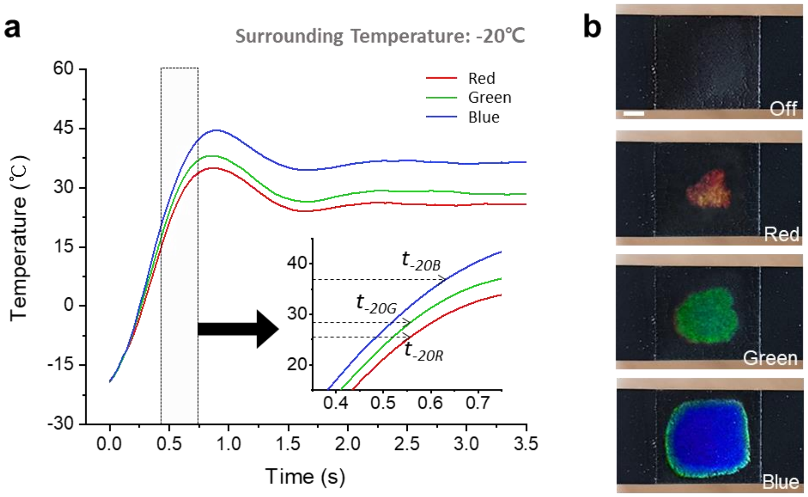

Supplementary Fig. 11. Performance of the ATACS under the extremely low-temperature. a Temperature response profiles of the ATACS with active control at an ambient temperature of -20 °C, **b** Optical images of ATACS in off, red, green, blue color states. The scale bar is 2 mm.

Supplementary Note 4. Mechanical and chemical stability of the ATACS

1) Mechanical stability

Peeling strength is measured by attaching an adhesive tape on the electrode composed of cPI-AgNW-cPI and the electrode composed of AgNW-cPI and pulling it off in the vertical direction. (Supplementary Fig. 12) When AgNW is covered with cPI, since the adhesive tape cannot directly contact AgNW, the adhesion force between the cPI films and the tape is measured as the peeling strength. However, if AgNW is exposed on the surface, the adhesive tape-AgNW or AgNW-cPI, whichever has the weaker adhesion, is detached first in the peeling test. In the Supplementary Fig. 12a, during the peeling test, the AgNW was separated firstly from the cPI film, and that indicates the adhesion strength of the AgNW-cPI is weaker than that of the cPI-tape. The peeling strength graph was shown in Supplementary Fig. 12b. The peeling strength of the cPI-AgNW-cPI composite averaged 1.46 N/mm during testing, and the AgNW-cPI averaged 0.44 N/mm. Therefore, it was confirmed that ATACS' AgNW heater is not affected by external physical damage because it is embedding in cPI.

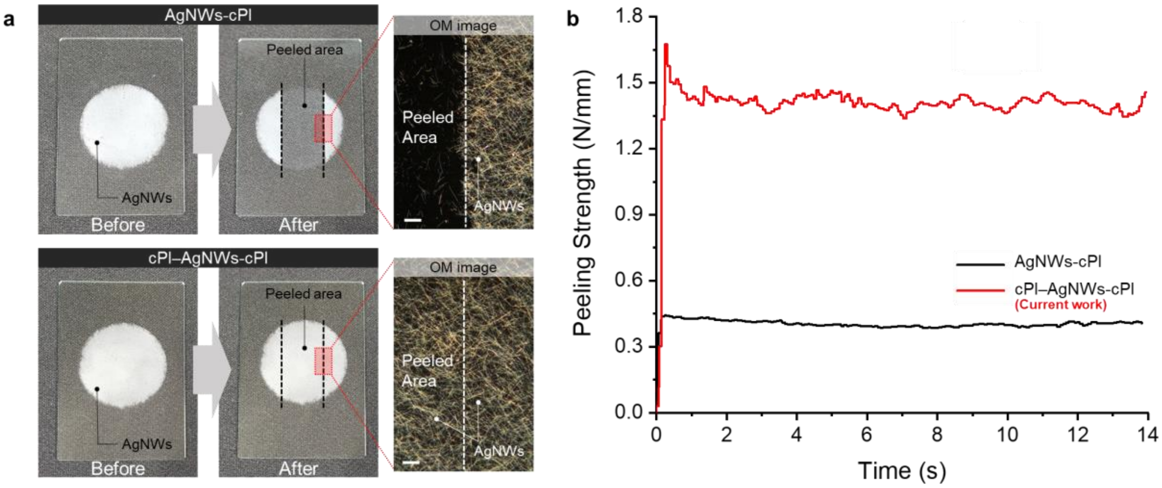

Supplementary Fig. 12. Peeling test of AgNW heater. **a** Digital and optical microscope images of before and after peeling test. The scale bar is 20 μm. **b** Peeling strength of AgNW heater covered with cPI is a higher value (average peeling strength: 1.45 N/mm) than AgNW heater without covering cPI layer (average peeling strength: 0.44 N/mm).

2) Chemical stability

Enhancement in chemical stability by cPI cover later is valid even in the presence of environmental chemicals. As an extreme example, the effect of H_2O_2, which is known to dissolve Ag efficiently, is tested on the AgNW-cPI (un-covered AgNW) and cPI-AgNW-cPI (cPI covered AgNW) composites. AgNW-cPI and cPI-AgNW-cPI electrodes are prepared at high densities, and half of each electrode is immersed in a 12.5% H_2O_2 aqueous solution for 100 seconds, followed by deionized (DI) water cleaning. The microscopic images of the electrodes are displayed inset image of Supplementary Fig. 13a. After the short contact with the H_2O_2 solution, the AgNW-cPI electrode is immediately degraded with a large amount of bubbles generation, and the immersed AgNWs are mostly removed from the cPI substrate. In contrast, for the cPI-AgNW-cPI electrode,

there is no perceptible change as presented in the microscope image in Supplementary Fig. 13a. This result implies that the cPI cover layer successfully protects the AgNW against the attack of the corrosive chemicals and therefore maintains its performance even at such harsh conditions in which the AgNWs are dissolved instantly. The electrical resistance change upon the exposure to H_2O_2 solution also confirms the advantage of cPI coating as shown in Supplementary Fig. 13a. Supplementary Fig. 13b shows the SEM images of the AgNW-cPI electrodes after the end of the chemical corrosion test. the AgNW is totally dissolved, and the only negligible residue is found.

In addition, we tested the chemical durability of the electrode in an environment with a humidity of 80% and a temperature of 80 degrees (80-80 environment), simulating an extreme environment. AgNW-cPI (un-covered AgNW) and cPI-AgNW-cPI (cPI covered AgNW) composites were exposed to an 80-80 environment and electrical resistance was measured every minute. (Supplementary Fig. 14) cPI-AgNW-cPI showed stable performance in resistance for more than 1,000 min as cPI cover layer protects AgNW from surrounding high temperature and high humidity environment. In contrast, AgNW-cPI showed an exponential increase in resistance over time due to direct exposure to moisture and high temperatures, and the electrical performance quickly degraded. Therefore, these two results clearly validate that the cPI-AgNW-cPI electrode is well-protected by the cPI cover layer to achieve highly enhanced chemical stability.

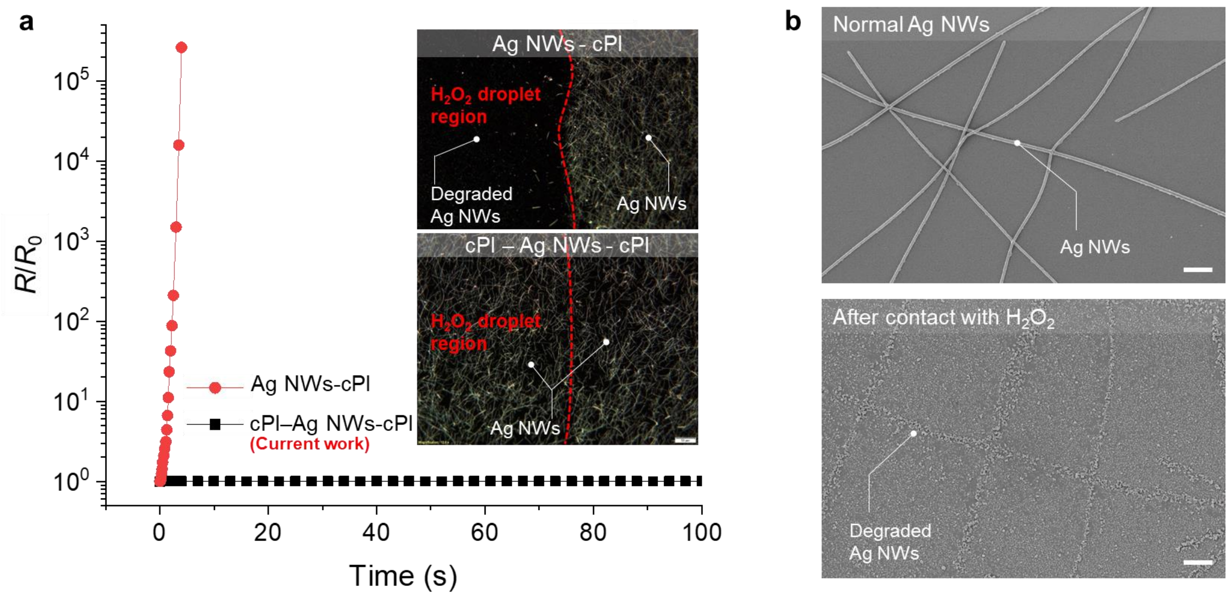

Supplementary Fig. 13. Chemical stability test of the AgNWs heater under the H_2O_2 condition. **a** electrical resistance changes of the AgNW-cPI (un-covered AgNW) and cPI-AgNW-cPI (cPI covered AgNW) electrode under H_2O_2 environment. **b** SEM image of AgNWs before and after H_2O_2 treatments.

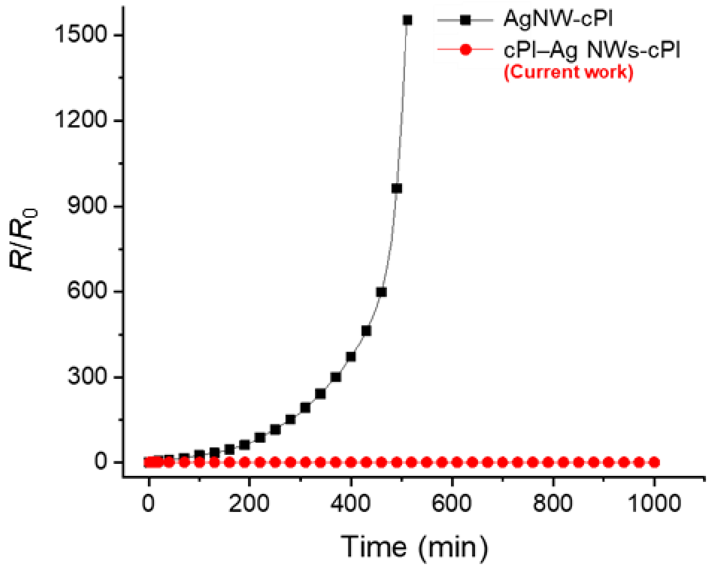

Supplementary Fig. 14. **The results of the 80-80 test on the AgNW-cPI and cPI-agNW-cPI composite**. The graph shows electrical resistance changes of the AgNW-cPI and cPI-AgNW-cPI electrodes under relative humidity 80% and 80 ˚C conditions.

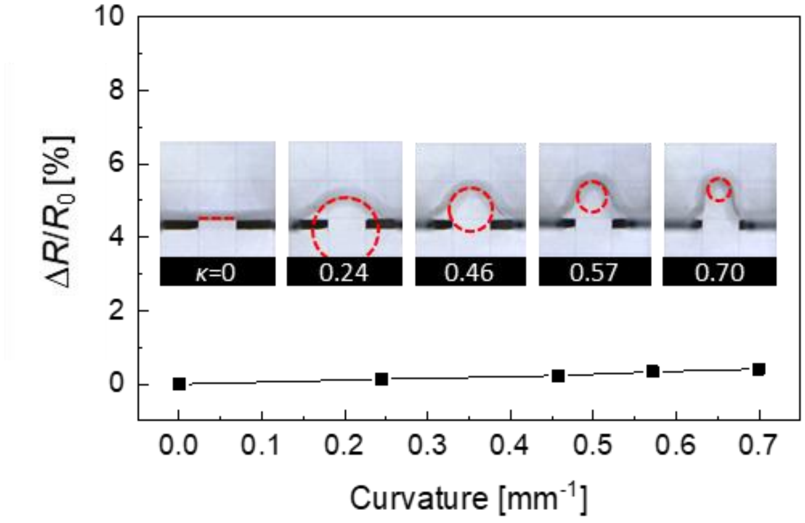

Supplementary Fig. 15. Mechanical stability test of the ATACS. Electrical resistance depending on various bending curvatures of the ATACS. The graph shows only 0.5% electrical resistance change of the ATACS at κ=0.65 mm^{-1}.

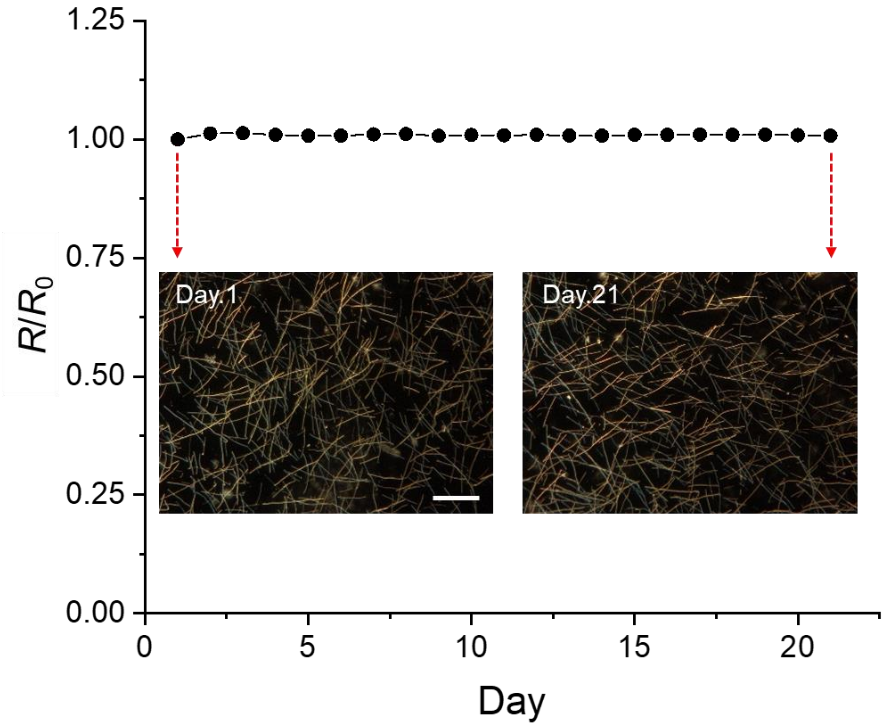

Supplementary Fig. 16. Long-term durability test of ATACS heater electrode. Insets are optical microscope images of the AgNW electrode. The scale bar is 40 μm. The electrical resistance changes of the ATACS electrode exposing to the air at room temperature for 21 days were measured every day. There was no significant change in the resistance of the ATACS electrode during the 21 days, and as a result of observing the electrode with an optical microscope, it was confirmed that there was no degradation in the electrodes.

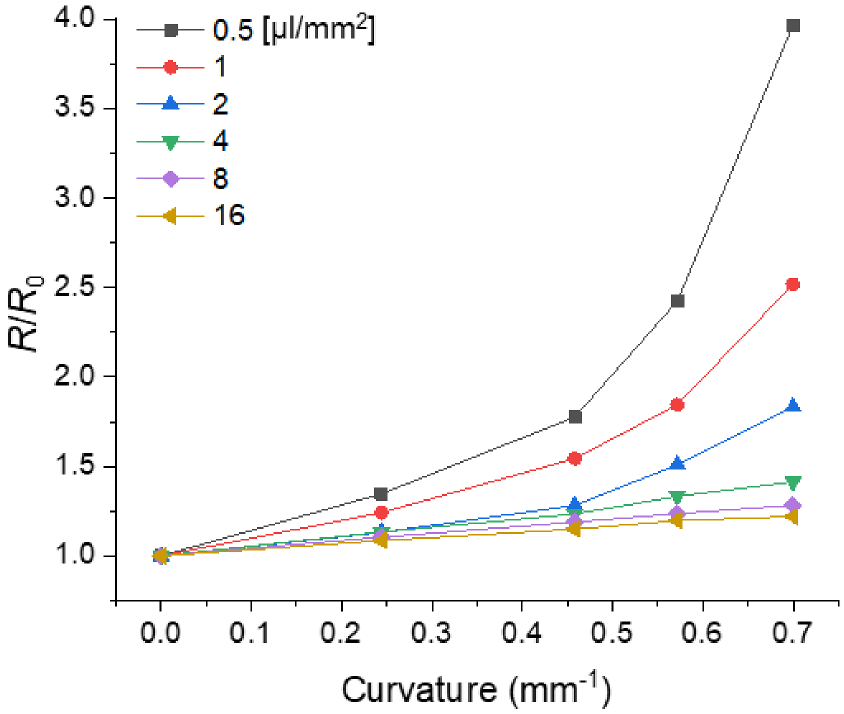

Supplementary Fig. 17. Electrical resistance sensitivity of ATACS with various AgNW solution densities. The graph shows electrical resistance change according to various curvatures (0.1 to 0.7 mm^{-1}) of the electrodes which are composed of various nanowire concentrations. (0.5 to 16 μl/mm^2) At a curvature of 0.7, the electrical resistance increases 396 % in electrode with 0.5 ul/mm^2 AgNW concentration and 24 % in electrode with 16 ul/mm^2 concentration. Because the probability of disconnection between nanowires due to mechanical deformation increases with low nanowire density, [69] the electrical resistance changes larger than electrode with high nanowire density. Therefore, by controlling the appropriate nanowire concentration, the electrical sensitivity could be modulated.

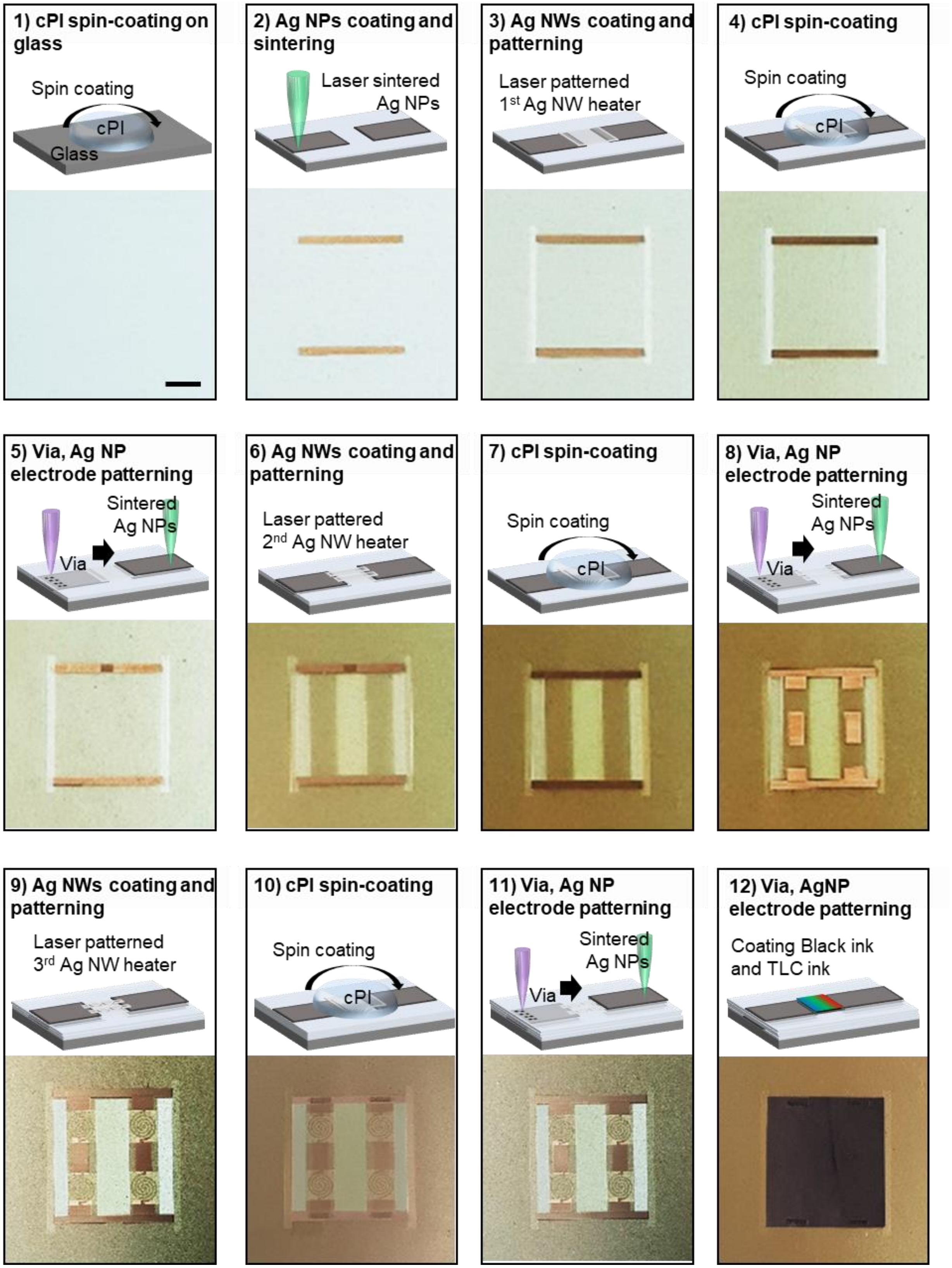

Supplementary Fig. 18. Schematics of the multi-layered ATACS fabrication 1) cPI varnish is

coated on the glass substrate using spin coater and then annealed with gradually increasing temperature to 300 ^{o}C. 2) Ag NPs inks are coated on annealed cPI layer and coated Ag NPs inks are selectively sintered to fabricate a base electrode. The scale bar is 5 mm. 3) Laser ablation process to patterned 1st Ag NW heater which is spay-coated on the Ag NP electrodes. 4) Spin-coating and annealing a cPI varnish as a cover layer on the 1st Ag NW heater. 5) Laser ablation on the target area to form electrical connection holes and using the laser sintering process, the Ag NP electrodes are formed which has an electrical connection between the bottom Ag NP electrodes and the upper one. 6) ~ 11) The procedure of 1) ~ 5) is repeated twice to fabricate vertically stack Ag NW heater with various designed patterns. 12) Spin coating black ink and TLC ink sequentially. Then, trimming the multi-layered ATACS and external readout electrode wiring.

Supplementary Note 5. Theoretical study and experiment for the evaluation of the multi-layered ATACS' thermal performance

First of all, the uniformity of heat propagation is rarely affected by the stacked AgNW layers. The theoretical maximum value of the thermal conductivity of the AgNW region consisting of cPI (k = 0.12 W/m·K at STP) and silver (k = 429 W/m·K at STP) is achieved when all silver grains are arranged perpendicular to the cross-section through which the heat passes as depicted in Supplementary Fig. 19. According to a rough calculation based on the amount of AgNWs used for one heater layer, the volumetric ratio of silver in the AgNW region is less than 2 % (Supplementary Fig. 20), hence the maximum thermal conductivity of the AgNW region is 8.70 W/m·K. A heater layer consists of ~ 0.2 μm of an AgNW region and ~ 3 μm of a pure cPI, and the overall thermal conductivity is 3.99×10^4 W/m^2·K, while that of 3.2 μm of a pure cPI film is 3.75×10^4 W/m^2·K. The difference is only about 6.6 % even at this extreme assumption, and it is a significantly small figure considering that the thermal conductivity of silver is about 3,500 times larger than that of cPI. We also conducted an experiment to verify the theoretical analysis above. Supplementary Fig. 21 presents the digital photographs together with the IR image of the TLC layer when the 1st layer is activated, which shows two features: (i) The temperature at the overlapped region is dropped pointedly, which in turn provides different colors (G→R) at the corresponding region. (ii) The temperature rises spread in the vertical direction to yield an unwanted color change in the vicinity. It is also predictable that these problems will become more pronounced once the number of layers is increased. On the contrary, these phenomena are largely suppressed when the metallic films are substituted by the AgNW percolation network, enabling an uninterrupted display of the patterns in each layer as we intended. Therefore, the degree to which the temperature distribution is

distorted by the silver nanowires is likely to be insignificant, and it seems unnecessary to consider the distortion of the pattern in the process of stacking the heaters.

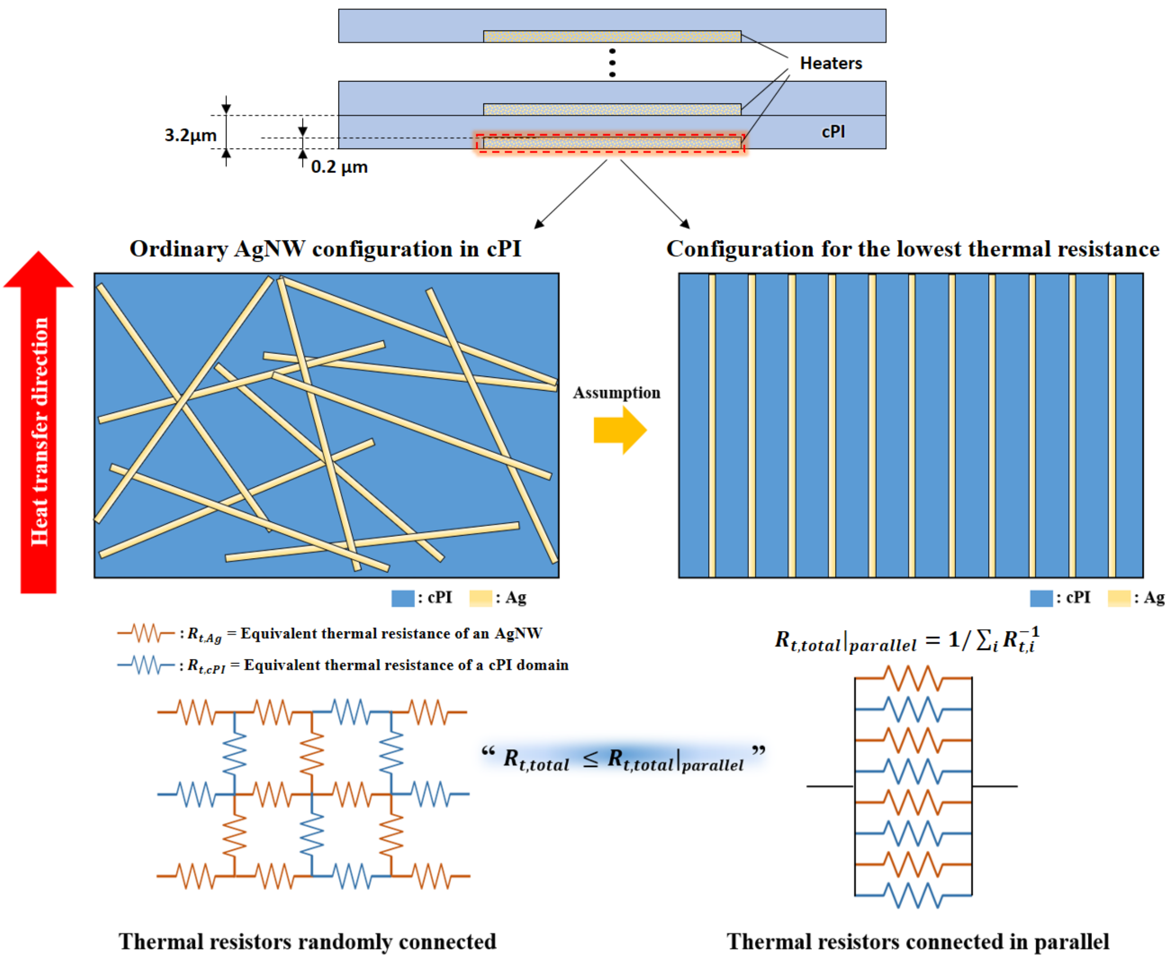

Supplementary Fig. 19. Schematics of the AgNW heater configuration with ideal maximum thermal conductivity. A parallel arrangement of AgNWs to the heat transfer direction minimizes the thermal resistance of the heater.

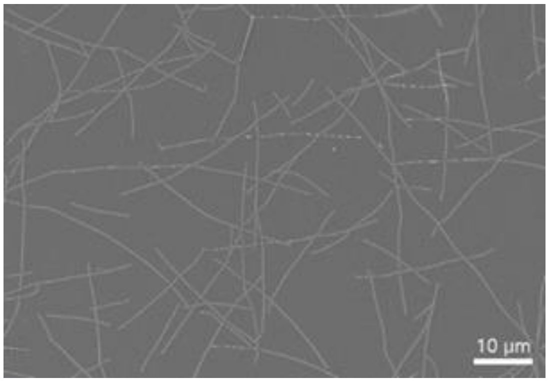

Supplementary Fig. 20. SEM image of AgNW-cPI layer to calculate the areal ratio of AgNW.

The area coverage is 1.667%.

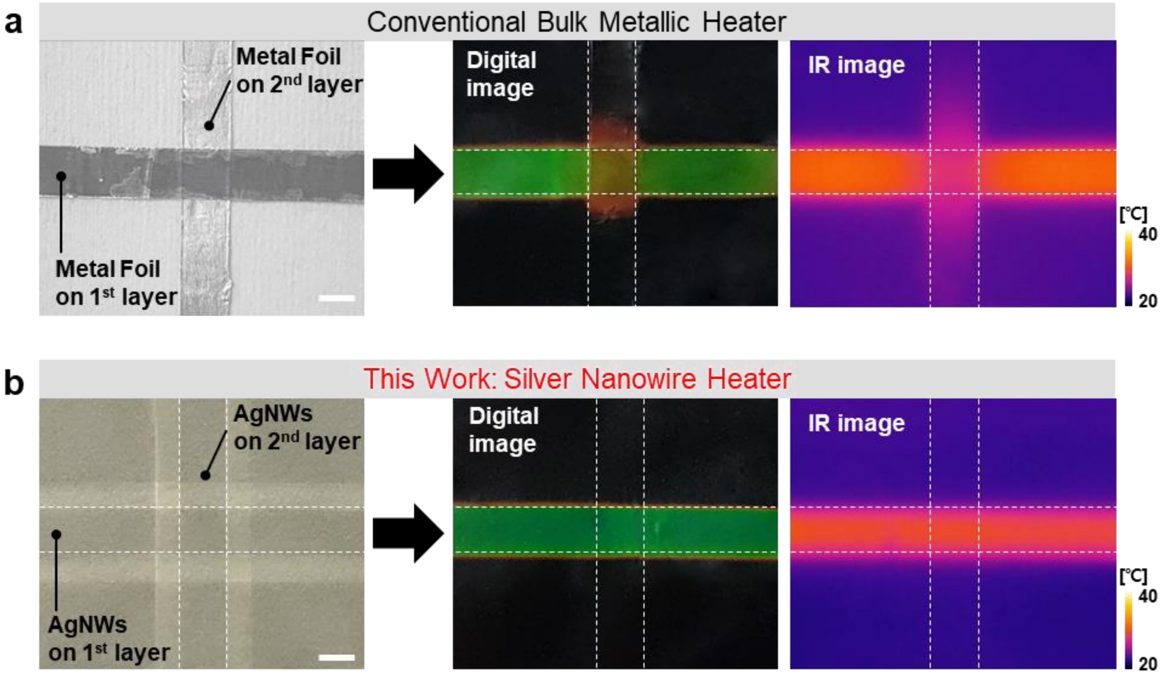

Supplementary Fig. 21. Comparison of thermal transparency between conventional bulk metal stacked heaters and nanowire stacked heaters. a Thermal conduction through the bulk electrode on another layer induced undesired coloration on the surface. The scale bar is 5 mm. **b** The AgNWs heater has better thermal transparency between layers than bulk heater configuration and shows accurate coloration. The scale bar is 5 mm.

Secondly, we conducted a frequency domain simulation (Comsol Multiphysics, Comsol Inc.) to evaluate how the number of the heater layer affects the performance of the temperature control i.e., how sensitively the temperature of the heater responds to a given signal. Supplementary Fig. 22a is a cross-section of the stacked heaters, and the bottom-most heater highlighted by red is generating joule heat with sinusoidal power input according to frequency. The frequency range of the input signal is from 0.1 Hz to 100 Hz, and the amplitude of the wave is fixed to 1.0 x 10^{10} W/m^3 which corresponds to the order of magnitude of the maximum operation power density for ATACS. The temperature response is measured at the midpoint of the upper boundary, and the convection coefficient of the air is assumed to be 30 W/m^2K, an ordinally used value for the standard atmospheric condition. To reduce the computational load, thermal properties (thermal capacitance and thermal conductivity) of the AgNW are neglected, since the AgNW with the small volume ratio to cPI seems to have only a slight influence on the overall thermal conductivity of in the heater region as discussed above.

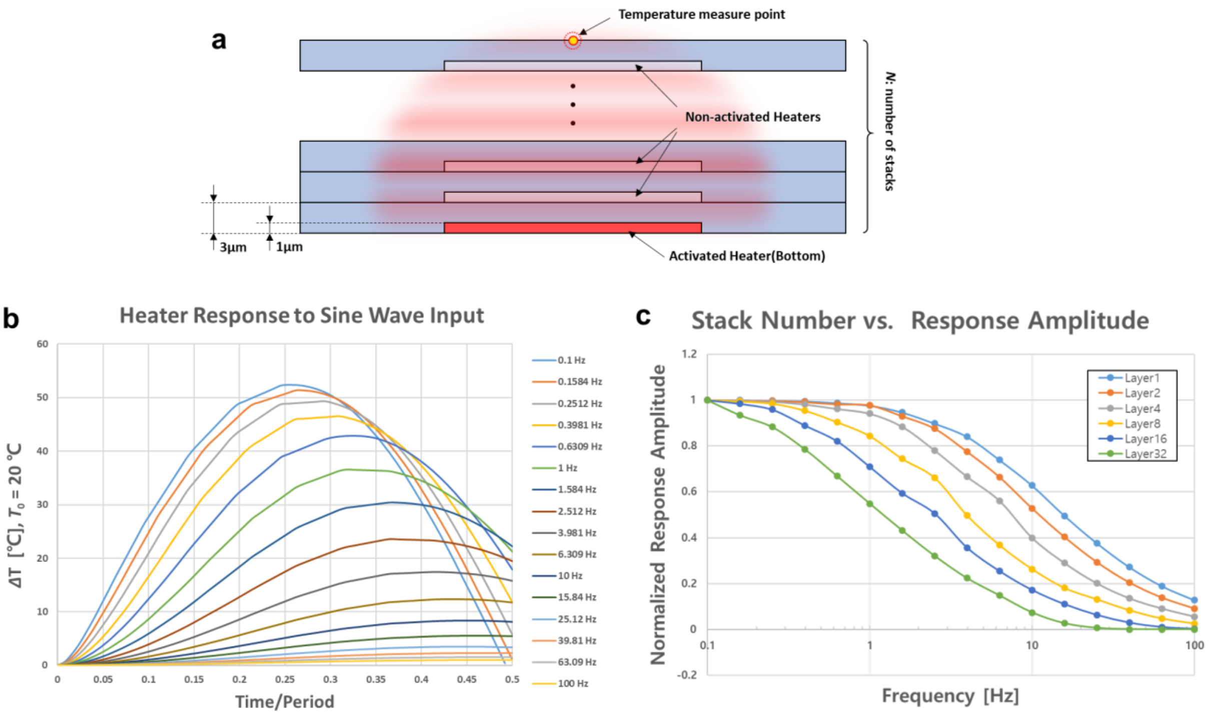

Supplementary Fig. 22. Numerical simulation to evaluate how the number of the heater layer affects the performance of the temperature control. a Simulation setup for the multi-layered ATACS **b** Temperature response of 16-layer ATACS. **c** Normalized amplitude of the temperature response according to heater frequency and the number of stacked heaters

Supplementary Fig. 22b shows magnitude attenuation and phase lag of temperature response for 16-layer ATACS with increasing heater frequency. Compared to the 0.1 Hz heater input, the maximum temperature is halved around the 2.512 Hz heater input, and the phase is delayed more than 1/4 period at 100 Hz input. Since the instantaneous maximum power is limited by the specification of the power source and the power driver module, the decrease in response magnitude at high-frequency ranges is practically difficult to be compensated for by the additional power input. Consequently, the controller-heater system loses control under high-frequency disturbance, leading to the unstable and fluctuating coloration of ATACS. The amplitude of the response for a

given heater frequency also decreases as the number of stacked heater layers increases. Supplementary Fig. 22c indicates the normalized response amplitudes (NRA) according to the heater frequency and the number of stacked heater layers (*N*), where the amplitudes are normalized by the lowest frequency input for each *N*. The cutoff frequency, where the NRA decreases to 50% of the saturated response, declines as *N* increases. Since the controller designed in this study operates at 30 Hz, the temperature response at this frequency may be considered as a criterion for the performance of the controller. Compared to when *N*=1 (NRA = 0.34), the NRA with *N* =8 is 3.34 times lower while the NRA with *N* =32 is at least 50 times lower. Though there are many factors to consider before the selection of a proper power diver module, we expect the NRA attenuation of less than 10 can be covered by the power margin of the ordinary low-cost power driver module. On the contrary, even if the NRA attenuation may be compensated by enhancing the specification of the power source, the large difference in NRA when *N* =32 would significantly increase the cost.

Supplementary Note 6. Camouflage accuracy of the ATACS

Camouflage accuracy can be defined as the similarity between the surrounding environment and the color and pattern of the object. [70] We conducted an additional study to analyze color accuracy, pattern accuracy, and color resolution of the ATACS and added text and figures to the supplementary material to further explain the content.

1) Color accuracy

Accurate coloration even in repeated operation is an important capability for the reliability of the camouflage skin and the effective crypsis. ATACS is a color-changing skin using a temperature-sensitive liquid crystal and the color of the ATACS is one-to-one corresponding to surface temperature with continuous transition characteristics. (Fig. 1f) This means that the color accuracy of the ATACS matches the accuracy of the temperature generated by the silver nanowire heater. We evaluated the performance of the heater for 1,000 repeated on-off cycles. At each target temperature of the TLC (Red: 25.5 ˚C, Green: 28 ˚C, blue: 36 ˚C), the heater of the ATACS shows the standard deviation during the cycle was within 0.1. (Supplementary Fig. 23) Also, to direct evaluation of the color accuracy, we measured Hue, Saturation, Value of the ATACS under high-repetition of the coloration. During and after 1,000 on-off cycles, ATACS shows the difference in Hue value within 1 % for Red, Green, Blue, showing uniform color generation ability without any hysteresis. (Supplementary Fig. 4) Therefore, the one-to-one correlation between temperature and color, the performance of the silver nanowire heater with low error value under high repetition, and the stable coloration ability of the ATACS show that the ATACS has good color accuracy.

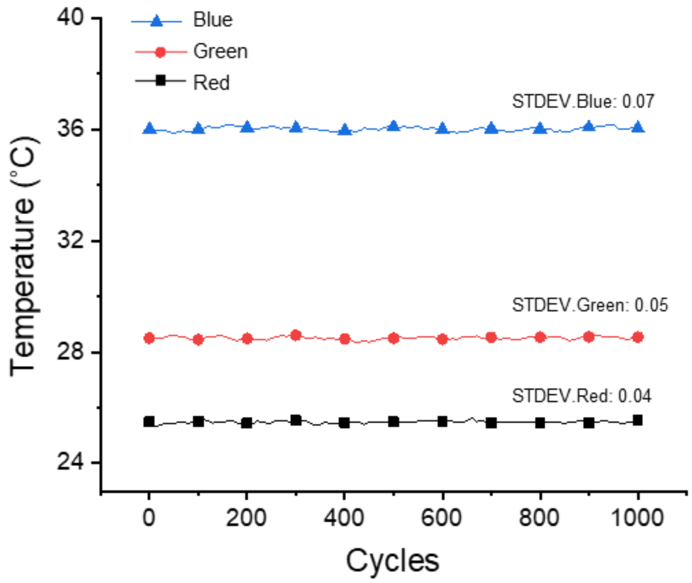

Supplementary Fig. 23. The on-off cyclic test of the silver nanowire heater. STDEV (Standard deviation) of the temperature is 0.04, 0.05, 0.07 for the Red, Green, Blue during 1,000 cycles.

2) Pattern accuracy

To evaluate the pattern accuracy of the ATACS, we compared the similarity between the S-shape image, the pixelated image to imitate it, and the ATACS with S-shape pattern. In order to imitate the original S-shape, we implement various pixelated images with different pixelation levels. Quantitative pattern similarity could be confirmed by comparing the area difference where the simulated pattern and the original shape did not match. (Supplementary Fig. 24)

The ATACS shows a similar area difference with the 64x64 pixelated image which has 625μm unit pixel size. However, whether the pattern accuracy is increased as the pixel size decreases, the complexity of the system also increases. In order to control the N x N heater array, N^2 signal nodes are required (Supplementary Fig. 25a), and even if a circuit using additional diodes, at least 2N signal nodes are required. (Supplementary Fig. 25b) However, the ATACS requires the number of signal nodes as much as the number of patterns, it is possible to implement a similar level of pattern

similarity to a 64x64 image with only one signal node.

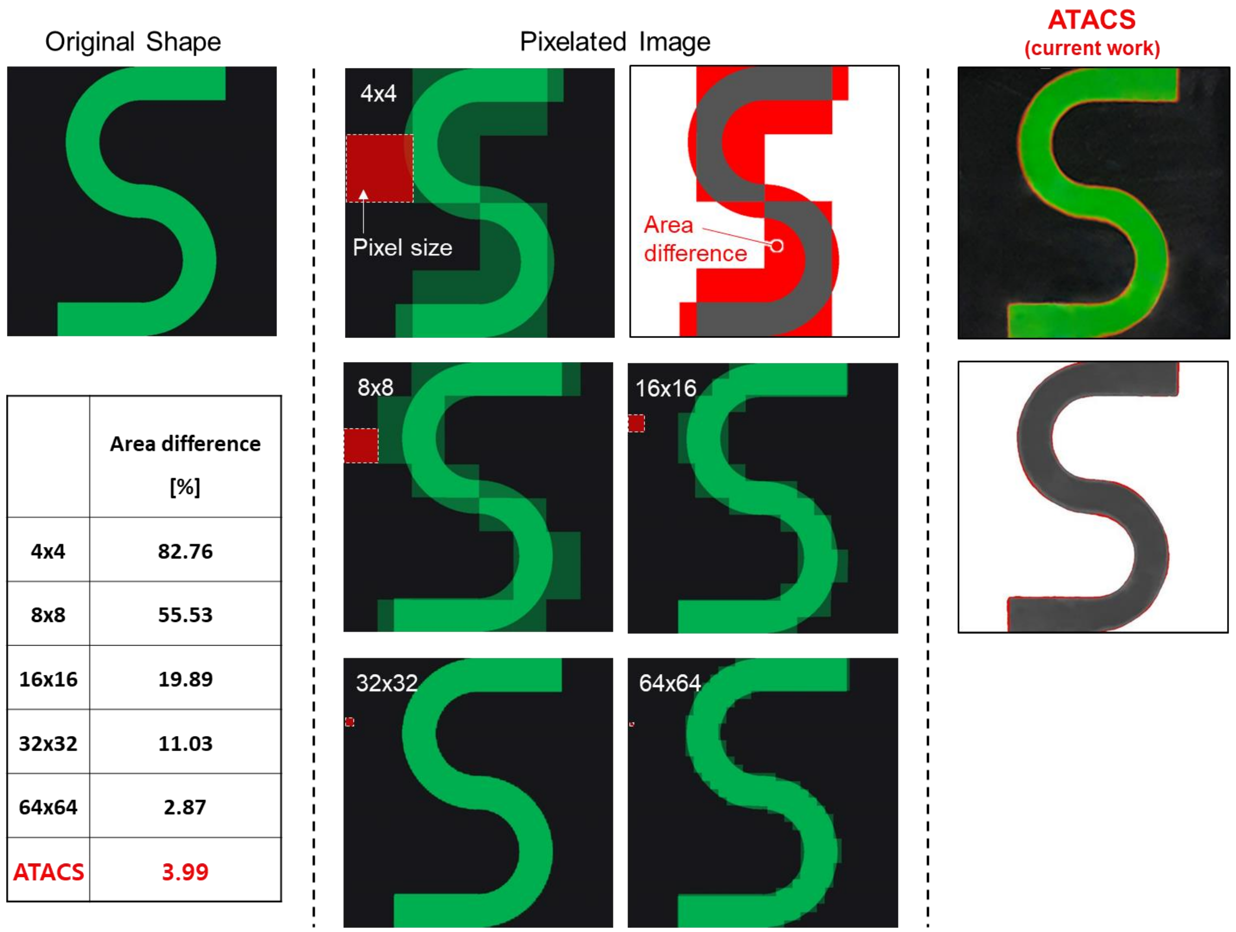

	Area difference [%]
4x4	82.76
8x8	55.53
16x16	19.89
32x32	11.03
64x64	2.87
ATACS	3.99

Supplementary Fig. 24. Pattern accuracy evaluation. The area difference between the original S-shape and pixelated image is 82.76 % for 4x4 pixelation, 55.53 % for 8x8, 19.89 % for 16x16, 11.03 % for 32x32, 2.87 % for 64x64, and 3.99 % area difference for the ATACS. The scale bar is 5 mm.

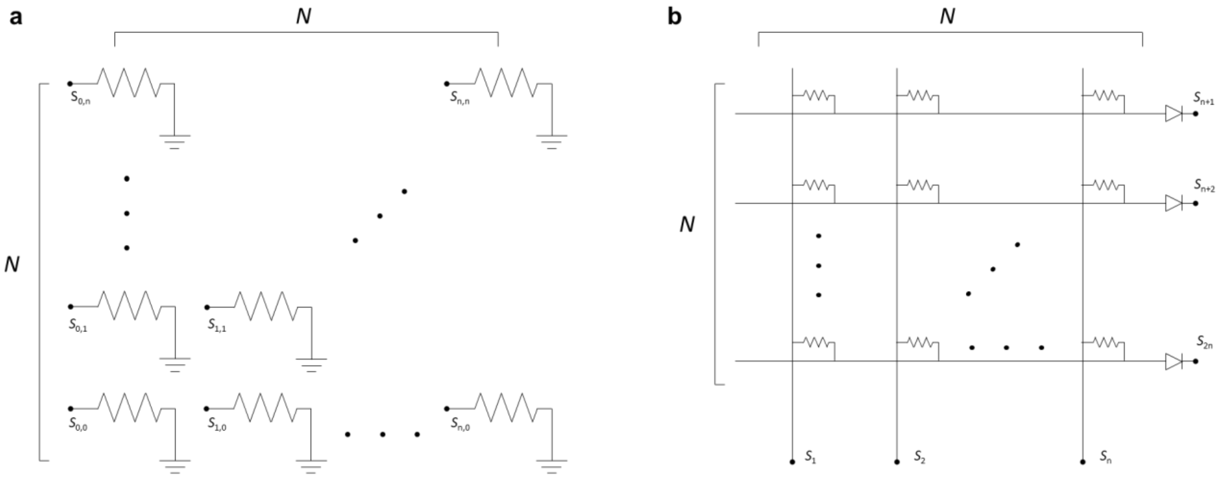

Supplementary Fig. 25. Schematics of *N* x *N* heater array circuit. a *N* x *N* heater array without diodes. **b** *N* x *N* heater array with additional diodes.

3) Color Resolution

In order to analyze the color resolution of the ATACS, we studied on the minimum dimension that the pattern expressed in the ATACS can be completely visually distinguished without being affected by the other surrounding patterns. 2 mm and 0.5 mm of single line heater are fabricated to investigate the color profile of the ATACS. (Supplementary Fig. 26) In Supplementary Fig. 26a, S26b, the ATACS at the red color state did not appear other colors on the outside of the heater line. However, because of thermal distribution in a plane direction of ATACS, 0.3 mm red color area over the heater boundary have appeared at the green color state, 0.3 mm red and 0.5 mm green area are appeared at the blue color state. The color resolution of ATACS was confirmed through a double line ATACS having a 0.5 mm line width at 0.5 mm intervals. From the case of blue, the color boundary is blurred at the corresponding dimension, indicating that the ATACS color resolution at room temperature is about 0.5 mm. (Supplementary Fig. 26c)

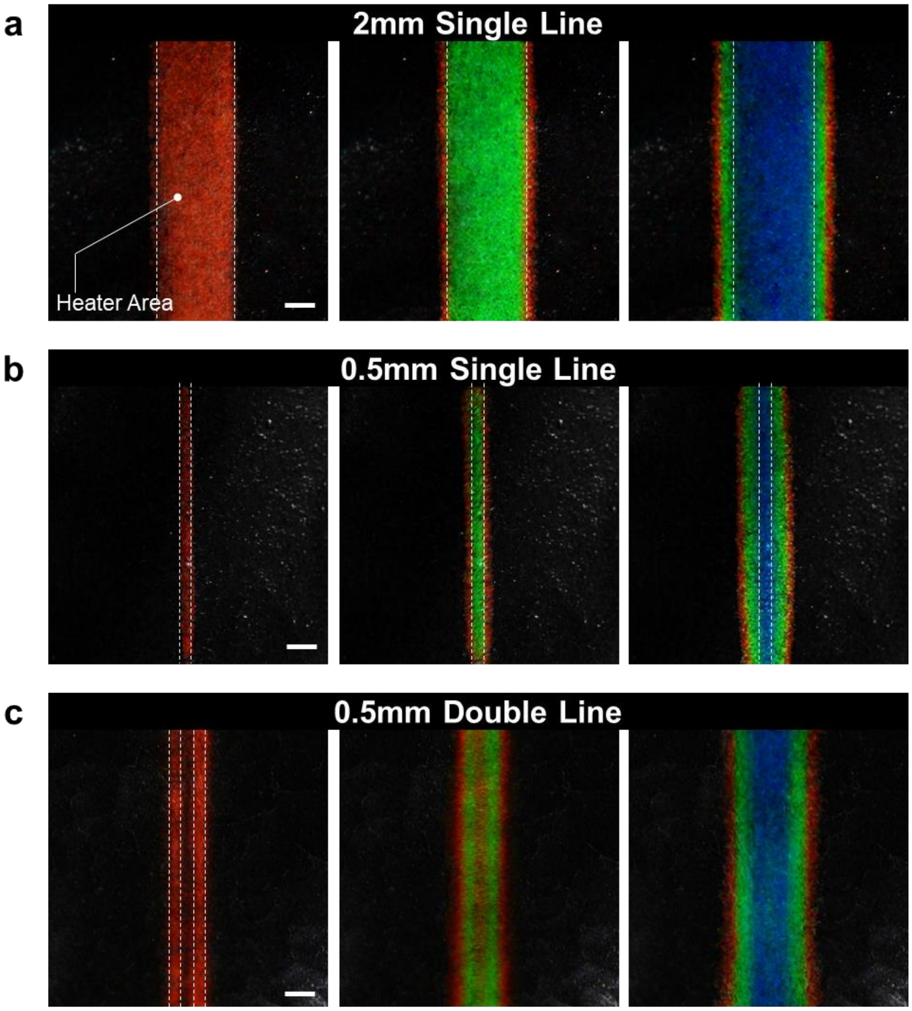

Supplementary Fig. 26. ATACS's feature resolution evaluation. a Single line patterned ATACS with 2 mm line width AgNW heater and image of the red, green, and blue color state. **a** Single line patterned ATACS with 0.5 mm line width AgNW heater and image of the red, green, and blue color state. **c** Double line patterned ATACS with 0.5 mm line width at 0.5 mm spacing AgNW heater. The scale bar is 1 mm.

According to the previous studies on the artificial camouflage device, the following table (Table S1) shows some representative examples regarding the artificial camouflage that employed the pixelated scheme to generate an arbitrary pattern. As a quantitative comparison, in the previous

study in Table S1, a camouflage skin is composed with a multi-pixel unit using a pixel size of at least 1 mm so far, and the ATACS of this study confirmed that much smaller features (~0.5 mm) could be expressed. (Supplementary Fig. 26) In addition, these pixelated artificial camouflage strategies which consist of independent pixels are brought additional issues which are about dead zone at the boundaries. 2D array configuration introduces dead zone, i.e. inactive area, at the borders of each pixel similar to the ones found in a charge-coupled device (CCD) and digital micromirror device (DMD). The dead zone acts as additional boundaries that hamper the camouflage performance not only for the uniform background, but also for complex backgrounds by bringing regular pattern that yields low complexity for visual perception.[71]

Authors	Journal	Year	Mechanism	# of pixels	Pixel size	Spectrum	Reference
Yu et al.	PNAS	2014	Thermochromic	16 X 16	~1 mm	Monochrome	72
Wang et al.	ACS Nano	2016	Plasmonic tuning	10 X 10	~5 mm	Visible	73
Xu et al.	Science	2018	Bragg stack modification	3 X 3	~2 cm	Infrared	74
Dong et al.	ACS Nano	2019	Strain-accommodating smart skin	8 X 8	~5 mm	Visible	75
Li et al.	Science Advances	2020	Reversible metal electrodeposition	3 X 3	~1 cm	Infrared, visible	76

Supplementary Table 1. Previous studies on artificial camouflage device that incorporates pixelated scheme to created spatially varying patterns

Supplementary Note 7. The critical dimension of AgNW patterning

The critical dimension of AgNW patterning was defined as the line width at the point where the resistance change caused by decreasing the line width deteriorated the performance. We set the figure of merit (FoM) for the electrode performance as follows, and electrical resistivity according to the line width is measured and compared.

$$FoM = \frac{R_0}{R}$$

Where, R = electrical resistivity of target sample,

R_0 = electrical resistivity of target sample with maximum line width (500 μm).

Electrodes coated with various amounts of AgNWs solution (100 μl to 600 μl) are patterned with different line widths (10 to 500 μm). Supplementary Fig. 27 shows the FoM of the electrode with 100 μl AgNWs solution decreased as the line width decreased, whereas, at concentrations above 500 μl, the electrode performance does not decrease even at the line width of 10 um. The critical dimension of AgNW patterning could be controlled according to the volume of the nanowire solution used, and it is possible to fabricate a stable heater electrode even with a small dimension of 10 μm.

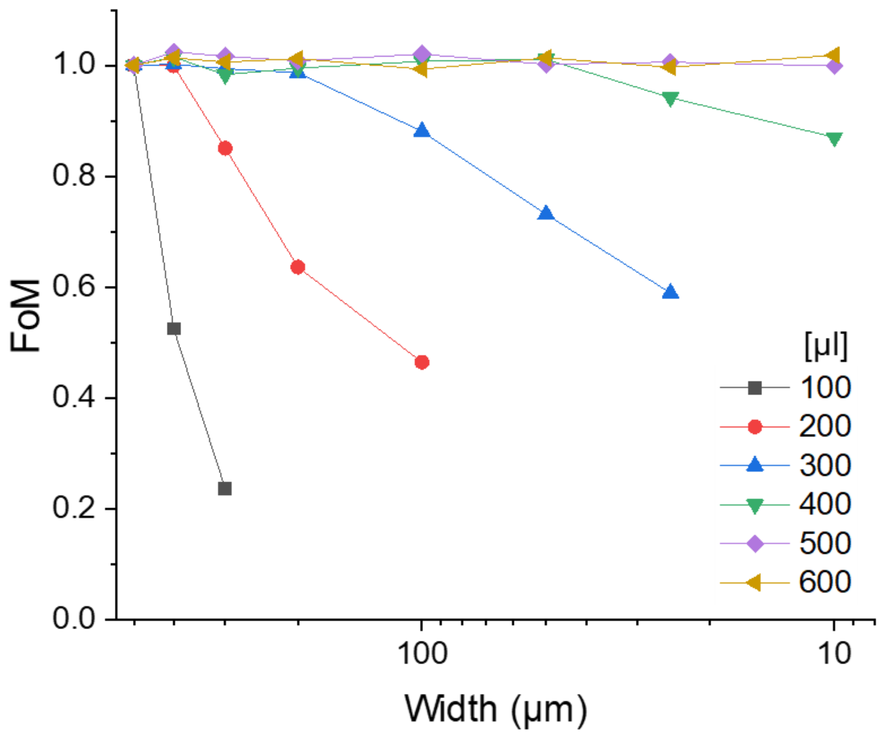

Supplementary Fig. 27. The critical dimension of AgNW patterning. FoM of AgNW electrode with various amounts of AgNW solution.

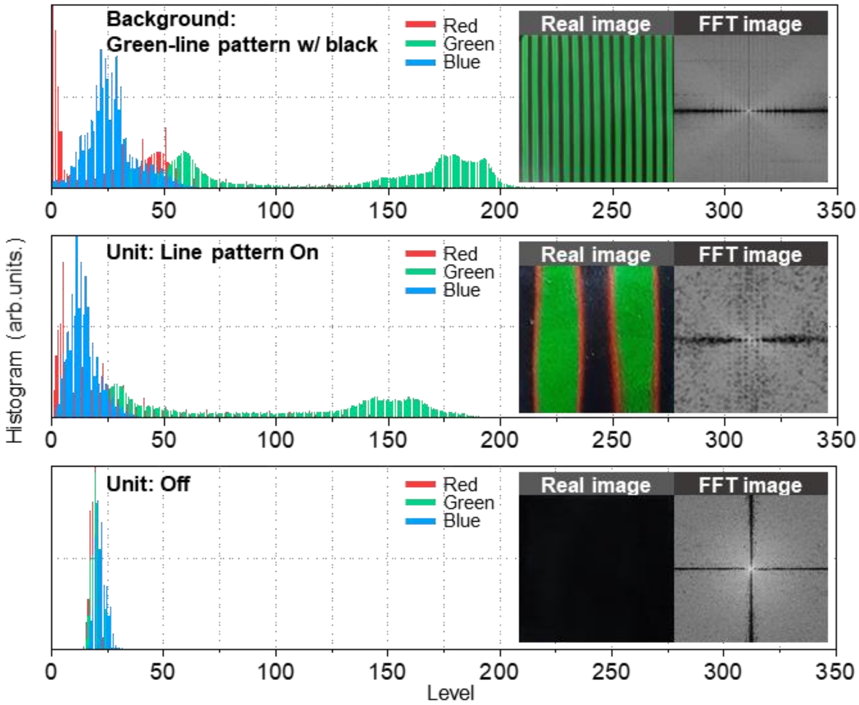

Supplementary Fig. 28. **Color and pattern of the ATACS similarity analysis.** RGB Histogram graphs and FFT images from each real image of inset (unit: off, unit: green line on, and green line background image). Comparing with data from the green line pattern background, the Unit of line pattern has a more similar color histogram profile and FFT pattern than off unit, implying the color and pattern of the line-pattern unit more similar to the background.

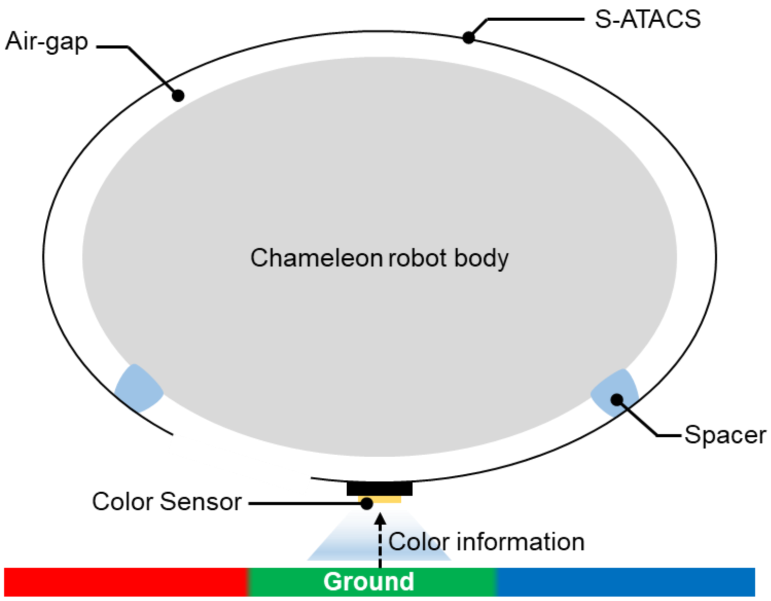

Supplementary Fig. 29. Schematics of ATACS attached to the chameleon robot body. Cross-sectional schematic image of the chameleon robot with S-ATACS. The color sensor is attached on edge of ATACS and adjacent to the ground. Also, for thermal insulation with the chameleon body, a spacer is placed on the chameleon robot body to secure the air gap.

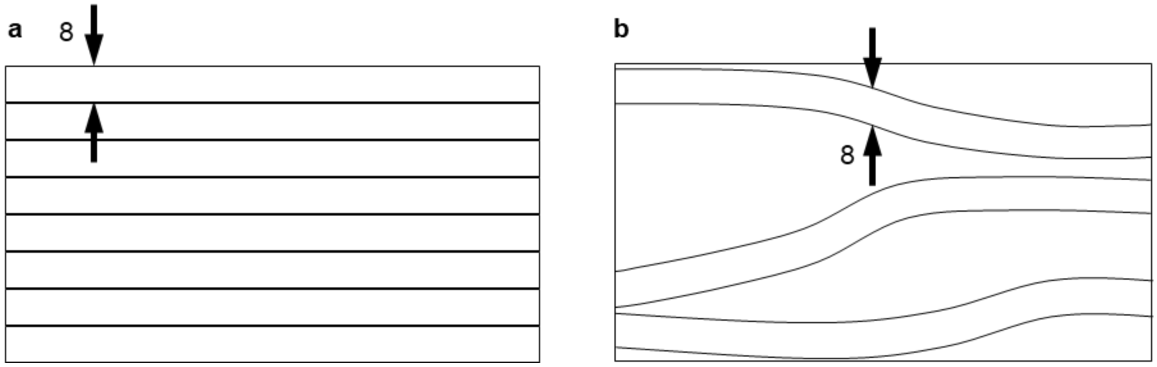

Supplementary Fig. 30. Specific dimensions of the heater patterns of ATACS on the chameleon model. a 1st layer of the ATACS for uniform pattern, **b** 2nd layer of the ATACS for the grass-like wavy pattern (unit: mm).

Supplementary Movies

Supplementary Movie 1. RGB Coloration performance of the ATACS. The ATACS heats up to target temperature corresponding to each target color i.e., red, green, and blue respectively. PID control algorithm enables quick response and robustness to various external disturbances.

Supplementary Movie 2. Real-time comparison of the ATACS with feedback control and without feedback control under external temperature disturbance. The PID control enables maintaining the temperature of the ATACS under strong external disturbance by approaching ice. Without the PID control, the ATACS is cooled down to lose its color by an ice chunk and recovers its colors slowly after removing the ice.

Supplementary Movie 3. Running ostrich demonstration of multi-layered ATACS. Three different ostrich postures heater, consisting of Ag NW and Ag NP electrode in series connection, are patterned by a laser process. The thickness of each ostrich-patterned heater layer is very thin (< 5um) compared to the TLC layer, hence an increase in the thermal capacitance by stacking is negligible. It shows fast color change response to input electrical signal, and the cycling activation of each independent ostrich pattern makes a simple animation of running ostrich.

Supplementary Movie 4. Camouflage demonstration of the chameleon robot with S-ATACS. The chameleon robot crawls through the red, green, and blue regions and changes its body color that matches the background. A color sensor under each ATACS recognizes the background color in real-time and transmits data simultaneously to a microcontroller which calculates optimum input energy to change the color of the target body.

Supplementary References

1. Collings, P.J. & Patel, J.S. Handbook of liquid crystal research. (1997).
2. Fergason, J.L. LIQUID CRYSTALS. *Scientific American* **211**, 76-85 (1964).
3. Hallcrest Handbook of thermochromic liquid crystal technology. (Hallcrest, 1991).
4. Tomke E. Glier, M.B., Maximilian Witte, Toru Matsuyama, Lea Westphal, Benjamin Grimm-Lebsanft, Florian Biebl, Lewis O. Akinsinde, Frank Fischerb, Michael Rübhausen Electrical and network properties of flexible silvernanowire composite electrodes under mechanical strain. *Nanoscale* **12**, 23831-23837 (2020).
5. Nokelainen, O., Brito, J.C., Scott-Samuel, N.E., Valkonen, J.K. & Boratynski, Z. Camouflage accuracy in Sahara-Sahel desert rodents. *J. Anim. Ecol.* **89**, 1658-1669 (2020).
6. Wu, J. et al. Enhanced Just Noticeable Difference Model for Images With Pattern Complexity. *IEEE Transactions on Image Processing* **26**, 2682-2693 (2017).
7. Yu, C. et al. Adaptive optoelectronic camouflage systems with designs inspired by cephalopod skins. *Proceedings of the National Academy of Sciences* **111**, 12998-13003 (2014).
8. Wang, G., Chen, X., Liu, S., Wong, C. & Chu, S. Mechanical Chameleon through Dynamic Real-Time Plasmonic Tuning. *ACS Nano* **10**, 1788-1794 (2016).
9. Xu, C., Stiubianu, G.T. & Gorodetsky, A.A. Adaptive infrared-reflecting systems inspired by cephalopods. *Science* **359**, 1495-1500 (2018).
10. Dong, Y. et al. Chameleon-Inspired Strain-Accommodating Smart Skin. *ACS Nano* **13**, 9918-9926 (2019).
11. Li, M., Liu, D., Cheng, H., Peng, L. & Zu, M. Manipulating metals for adaptive thermal camouflage. *Science Advances* **6**, eaba3494 (2020).
