# Supplementary material for: Biomimetic chameleon soft robot with artificial crypsis and disruptive coloration skin
Source: Nat Commun. 2021 Aug 10;12:4658. doi: 10.1038/s41467-021-24916-w (PMC8355336; doi:10.1038/s41467-021-24916-w)
Supplement: Supplementary file 2 — Description of Additional Supplementary Files [file 41467_2021_24916_MOESM2_ESM.pdf]

Description of Additional Supplementary Files

File Name: Supplementary Movie 1
Description: RGB Coloration performance of the ATACS.

File Name: Supplementary Movie 2
Description: Real-time comparison of the ATACS with feedback control and without feedback control, under external temperature disturbance

File Name: Supplementary Movie 3
Description: Running ostrich demonstration of multi-layered ATACS

File Name: Supplementary Movie 4
Description: Camouflage demonstration of the chameleon robot with S-ATACS
